# Hairy Root Cultures as a Source of Polyphenolic Antioxidants: Flavonoids, Stilbenoids and Hydrolyzable Tannins

**DOI:** 10.3390/plants11151950

**Published:** 2022-07-27

**Authors:** Janusz Malarz, Klaudia Michalska, Yulia V. Yudina, Anna Stojakowska

**Affiliations:** 1 Maj Institute of Pharmacology, Polish Academy of Sciences, Smętna Street 12, 31-343 Kraków, Poland; malarzj@if-pan.krakow.pl (J.M.); klaudiaz@if-pan.krakow.pl (K.M.); 2 National Technical University, Kharkiv Polytechnic Institute, Kyrpychova Street 2, 61002 Kharkiv, Ukraine; eco3557@gmail.com

**Keywords:** *Agrobacterium rhizogenes*, chalcone, ellagitannin, flavone, flavonol, isoflavonoid, proanthocyanidin, resveratrol, *Rhizobium rhizogenes*

## Abstract

Due to their chemical properties and biological activity, antioxidants of plant origin have gained interest as valuable components of the human diet, potential food preservatives and additives, ingredients of cosmetics and factors implicated in tolerance mechanisms against environmental stress. Plant polyphenols are the most prominent and extensively studied, albeit not only group of, secondary plant (specialized) metabolites manifesting antioxidative activity. Because of their potential economic importance, the productive and renewable sources of the compounds are desirable. Over thirty years of research on hairy root cultures, as both producers of secondary plant metabolites and experimental systems to investigate plant biosynthetic pathways, brought about several spectacular achievements. The present review focuses on the *Rhizobium rhizogenes*-transformed roots that either may be efficient sources of plant-derived antioxidants or were used to elucidate some regulatory mechanisms responsible for the enhanced accumulation of antioxidants in plant tissues.

## 1. Introduction

The interest in hairy root (HR) cultures as fast-growing, genetically stable axenic roots that are effective producers of both biomass and specialized plant metabolites started in the 1980s [1]. Since then, numerous culture systems have been established to produce compounds of economic value or potential economic interest to produce recombinant proteins to obtain transgenic plants of altered phenotype to obtain artificial seeds and perform metabolic engineering of plants [1,2,3,4,5,6]. The ability of *Rhizobium rhizogenes* (also known as *Agrobacterium rhizogenes*) [7] to transfer and permanently incorporate a foreign genetic material into the plant genomic DNA has also been used to study the regulatory mechanisms of plant metabolism [6,8,9]. Additionally, the enzymatic activity of HRs may be utilized for biotransformation and phytoremediation processes [10,11,12,13,14].

In contrast to other fast-growing types of plant tissue cultures, HRs are usually cultivated in nutrient media with a reduced concentration of macronutrients and without the addition of growth regulators that lower the costs of biotechnological processes. Several strategies were employed to scale up HR cultures from the laboratory to industrial applications, including various bioreactor designs [15,16], e.g., airlifts, bubble columns and nutrient mist, etc. The production of biologically active natural products and proteins by HR cultures has been a subject of numerous patents and shows potential for successful industrial applications. 

Until now, the main limitations that hamper the industrial use of HR cultures are still unsatisfactory upscaling results (the need for large-volume bioreactors) and unsatisfactory cost/profit ratios.

Nearly every class of plant-specialized metabolite includes some compounds of antioxidative activity [17,18,19,20], but the most effective antioxidants, acting by direct free-radical scavenging, originate mostly from phenolics and terpenoids [21,22]. Numerous natural products known as antioxidants are also recognized as potent chemopreventive and anticancer agents, influencing various intracellular signaling pathways [23,24]. Antioxidants of plant origin may also be useful as protective factors against dysfunctions of nervous system connected with aging [25].

Plant metabolites with antioxidant activity, which are the constituents of widely grown food plants, can be isolated from post-production waste or obtained as side products in the process of food manufacturing [26,27,28,29]. Some natural antioxidant products are a common occurrence and their isolation from either cultivated or wild plants seem to be the optimal solution. The yields of these natural products may be improved by bioengineering using the transformation by *R. rhizogenes*. In the case of threatened or overexploited plant species, HR cultures offer an opportunity to produce rare and valuable compounds with no harm to the natural habitats of the plant.

The present review is based on the results published in journals indexed in the Web of Science and Scopus databases. Due to the large number of studies dealing with polyphenolic metabolites of HRs, we decided to limit the scope of the review to flavonoids, stilbenoids and hydrolyzable tannins.

## 2. Polyphenolic Antioxidants in HRs

**Figure 1:**
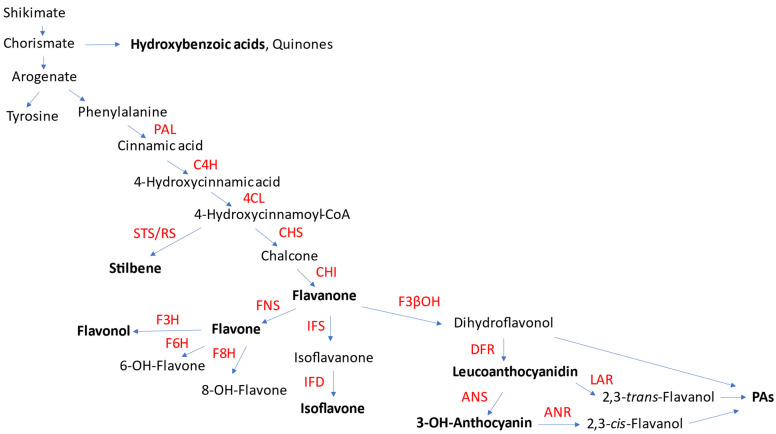
Simplified scheme of biosynthesis of selected plant polyphenols and positions of biosynthetic enzymes mentioned in the review (PAL—phenylalanine ammonia lyase; C4H—cinnamate 4-hydroxylase; 4CL—4-coumarate:CoA ligase; STS—stilbene synthase; RS—resveratrol synthase; CHS—chalcone synthase; CHI—chalcone isomerase; FNS—flavone synthase; IFS—isoflavone synthase; F3βOH—flavanone 3-β-hydroxylase; F3H—flavone 3-hydroxylase; F6H—flavone 6-hydroxylase; F8H—flavone 8-hydroxylase; IFD—isoflavone dehydratase; DFR—dihydroflavonol 4-reductase; ANS—anthocyanidin synthase; ANR—anthocyanidin reductase; LAR—leucoanthocyanidin reductase). Hydroxybenzoic acids group include gallic acid, a precursor of hydrolyzable tannins; PAs—proanthocyanidins).

Phenolics are one of the largest classes of plant-specialized metabolites. They all originate from the shikimate pathway leading to aromatic amino acids phenylalanine and tyrosine and also share the phenylpropanoid pathway (Figure 1). The compounds are divided into several subclasses, including flavonoids, tannins, stilbenoids, lignans, hydroxycinnamates and phenylethanoids, to mention a few of the most popular subclasses.

### 2.1. Flavonoids

#### 2.1.1. Anthocyanins and Proanthocyanidins (PAs)

Numerous factors may influence anthocyanin and PA production by HRs. The most frequently studied were the effects of light, plant growth regulators, sucrose concentration and the *R. rhizogenes* strain used in the experiment. The overexpression or heterologous expression of genes-encoding transcription factors implicated in the regulation of flavonoid biosynthesis (especially members of *myb* gene family) or the overexpression of genes-encoding biosynthetic enzymes (*DFR*, *LAR*, *ANR*) are strategies often used to increase anthocyanins and PAs content in the HRs or transgenic plants regenerated from the roots.

**Figure 2:**
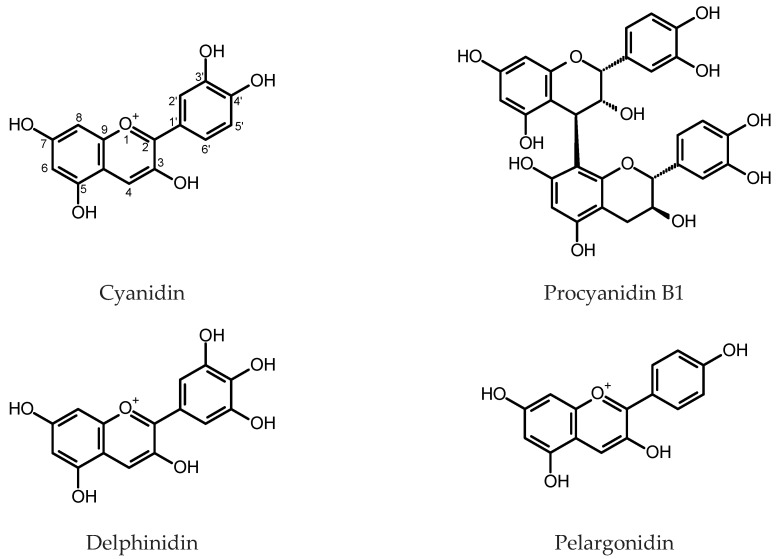
Chemical structures of selected anthocyanins and procyanidin B1.

Morris and Robbins [30] observed that the HRs of *Lotus corniculatus* L. (Fabaceae), maintained in the dark, accumulated insoluble tannins, which presented cyanin and delphinidin at a ratio like that found in the intact plant after hydrolysis. Supplementation with auxins reduced the content of tannins in the roots. On the contrary, in the HR cultures of *Leontopodium alpinum* Cass. (Asteraceae), the addition of 6-benzylaminopurine (BAP) to the culture medium 14 days prior to harvest increased the anthocyanins content [31]. An effect of auxin supplementation on anthocyanin contents in the HRs of Tartary buckwheat (*Fagopyrum tataricum* (L.) Gaertn., Polygonaceae) cultivar “Hokkai T10” was also studied [32]. Indole-3-butyric acid (IBA) added to a nutrient medium at a concentration of 4.92 µM, caused enhanced accumulation of cyanidin 3-*O*-glucoside and cyanidin 3-*O*-rutinoside in the roots (for structures, see Figure 2). The anthocyanin content reached 0.89 and 1.15 mg/g dry weight (DW), respectively, and was about three times higher than that found in the control cultures. 

Nishiyama and Yamakawa investigated effects of the culture illumination, composition of nutrient medium and sucrose content on the anthocyanin production in the HRs of *Ipomea batatas* (L.) Lam. (Convolvulaceae) [33]. They found that light and high sucrose concentration in the medium (5%) favored anthocyanin production. The beneficial effect of light on cyanidin 3-*O*-rutinoside accumulation was also observed in Tartary buckwheat HRs [34]. Motomori et al. [35] studied polyphenolics accumulation in the HRs of *Fragaria x ananassa* Duch. cv. Reikou (Rosaceae). In optimum conditions, the root culture produced up to 8 mg/g DW of procyanidin B-3, but the roots of the field-grown plant contained three times as much of the compound (24.2 mg/g DW).

The expression of full-length cDNA encoding dihydroflavonol 4-reductase (DFR) from *Antirrhinum majus* L. (common snapdragon, Plantaginaceae) in the HRs of *Lotus corniculatus* L. led to several high-producing clones. The maximum content of condensed tannins in the control roots was estimated as 0.62 mg/g fresh weight (FW), whereas the transgenic roots produced up to 1.06 mg/g FW of the compounds [36]. The proportions of subunits (procyanidin, prodelphinidin and propelargonidin) yielded on the hydrolysis of condensed tannins were also measured, and significant differences in propelargonidin accumulation were observed in the transgenic clones in comparison with the controls. The plasmid 121.Sn carrying the maize gene *Sn*, which is responsible for the transactivation of the anthocyanin pathway in different tissues, was introduced into the genomes of ten different plant species using the transformation with *R. rhizogenes.* Over 50% of the obtained *Medicago sativa* L. (Fabaceae) and *Lotus angustissimus* L. accession CPI 113587 HR clones showed pigmentation. On the other hand, the HRs of *Lotus corniculatus*, *L. japonicus* (Regel) K. Larsen and *L. angustissimus* accession CPI 113590 produced no pigment. The results suggested the transcription factor *Sn* derived from monocotyledonous plant may function in several dicot species [37].

The HRs of *Medicago truncatula* Gaertn. (Fabaceae) and *Vitis vinifera* L. (Vitaceae) were used to investigate the mechanisms of anthocyanin biosynthesis regulation by transcription factors encoded by the members of *myb* gene family [38,39,40,41,42,43,44,45]. The expression of *Arabidopsis* TT2 (Transparent Testa 2) transcription factor from the MYB family in the HRs of *M. truncatula* led to the induction of the genes for flavonoid and proanthocyanidin (PA) biosynthesis pathway and enhanced accumulation of PAs in the roots. The average soluble PAs content in *TT2*-carrying transformants was over ten times higher than that in the control HRs transfected with an “empty”vector. Insoluble PAs content in *TT2*-expressing roots was over 24-fold higher [38]. The induction of genes for PAs biosynthetic pathway was also true when the transcription factors: GmTT2A, GmTT2B or GmMYB5A were ectopically expressed in soybean (*Glycine max* L.) HRs [46]. Expression of *AtTT2* in chickpea HRs resulted in a high accumulation of soluble PAs (>1200 μg catechin equivalents (eq.)/g FW) [47]. MtPAR MYB transcription factor expressed in *M. truncatula* HRs dramatically increased soluble PAs content (up to 100-fold higher than that in the control roots) but did not influence insoluble PAs accumulation and diminished the anthocyanin content. Maximum content of soluble PAs reached 10 μM of (−) epicatechin eq. per g FW [40]. Li et al. [48] found that transgenic *M. truncatula* HRs that over-expressed *MtPAR* were characterized by enhanced accumulation of soluble PAs and diminished production of isoflavones. The role of MtPAR in the distribution of precursors to flavonoid, isoflavonoid, anthocyanin and PA pathways was emphasized by the same study. The HRs of *M. truncatula* overexpressing *MYB2* demonstrated suppression of the expression of genes-encoding dihydroflavonol 4-reductase (DFR1) and anthocyanidin synthase (ANS). Anthocyanin accumulation characteristic of wild-type roots was ceased in the *MYB2*-overexpressing roots [43]. Wild-type HRs of *M. truncatula* with the ectopic expression of MtTT8 transcription factor showed enhanced expression of the flavonoid biosynthesis pathway genes and elevated production of PAs and anthocyanins. Contents of anthocyanins (as cyanidin 3-*O*-glucoside eq.), soluble PAs (as epicatechin eq.) and insoluble PAs (as proanthocyanidin B1 eq.) in the *MtTT8*-expressing roots reached 600 μg/g FW, 27 μg/g FW and 180 μg/g FW, respectively [44]. Heterologous expression of *GhMYB36*—a gene for TT2-type MYB transcription factor from a tetraploid *Gossypium hirsutum* L. (Malvaceae), in the HRs of *M. truncatula* made it possible to achieve 100 μg/g FW of total soluble PAs (epicatechin eq.) and over 1600 μg/g FW of total insoluble PAs (proanthocyanidin B1 eq.) [44]. VvMybPA1 and VvMybPA2 are transcription factors involved in the regulation of PA biosynthesis in *V. vinifera* seeds, leaves and exocarp of young berries. The ectopic expression of the genes-encoding these transcription factors in the HRs of *V. vinifera* caused activation of the enzymes of the flavonoid pathway and enhanced accumulation of PAs (up to 8 mg/g FW). Moreover, the *VvMybPA1*- and *VvMybPA2*-expressing transformed roots started to produce PAs with trihydroxylated ring B [39].

The overexpression of VvMYBC2-L1 in grapevine HRs negatively affected PAs biosynthesis, as it can be expected for a member of subgroup 4 MYB transcription factors [41]. A similar effect was achieved in *V. vinifera* HRs by an overexpression of VvMYBC2-L3 [42]. Transgenic HRs of *Litchi chinensis* Sonn. (Sapindaceae), overexpressing *LcMYB1*, contained over 3 mg/g FW of anthocyanidins and nearly 15 mg/g FW of PAs. Wild-type lychee HRs accumulated about 5 mg/g FW PAs and minute amounts of anthocyanidins [49]. Regulatory functions of PpMYB10.1 and MYB 182 transcription factors in anthocyanidin biosynthesis were also investigated using peach and poplar HR cultures, respectively [50,51]. Expression of *Camellia sinensis* (L.) Kuntze-derived genes-encoding leucoanthocyanidin reductase (LAR) and anthocyanidin reductase (ANR) in the HRs of *M. truncatula* resulted in slightly elevated content of insoluble PAs. The ectopic expression of *CsLAR* enhanced the accumulation of anthocyanins, whereas *CsANRs* expressing roots tend to accumulate more soluble PAs than the control roots [52].

HRs of Tartary buckwheat were frequently used to investigate the effects of different factors on anthocyanins accumulation. Thwe et al. [53] examined seven wild-type *R. rhizogenes* strains to assess transformation efficiency and anthocyanin content in regenerated hairy root clones of *F. tataricum* cv. “Hokkai T10”. The R1000 strain, except for the highest transformation efficiency and growth rate of roots, provided high transcript levels for most genes of the flavonoid biosynthetic pathway and the highest contents of cyanidin 3-*O*-glucoside (800 µg/g DW) and cyanidin 3-*O*-rutinoside (2410 µg/g DW). A plant growth regulator—ethephon—at the concentration of 0.5 mg/L, significantly increased cyanidin 3-*O*-glucoside and cyanidin 3-*O*-rutinoside accumulation in hairy roots of buckwheat. Moreover, upregulation of the genes involved in flavonoid biosynthesis upon ethephon treatment was observed [54]. Overexpression of *FtMYB1*, *FtMYB2*, *FtMYB3* and *FtMYB-like* in the HRs of Tartary buckwheat up-regulated the genes of the phenylpropanoid biosynthetic pathway and enhanced anthocyanin production. *FtMYB18* overexpression negatively influenced anthocyanin biosynthesis in the roots [9,55].

The black or purple carrot (*Daucus carota* L. ssp. *sativus* var. atrorubens Alef, Apiaceae) belongs to the Eastern group of domesticated carrots and is cultivated and commonly eaten in India, Pakistan, Turkey and Afghanistan. In contrast to the Western orange carrots, the roots of the plant accumulated anthocyanins. Acylated anthocyanidin triglycosides produced by the plant, due to their chemical stability, are used as food colorants [56]. The HRs of black carrot were obtained by the infection of taproot and hypocotyl explants of black carrot inbred line “43” and taproot explants of cv. “Night Bird”. The maximum total monomeric anthocyanin content, spectrophotometrically measured, exceeded 3 mg/g DW and was found in the HRs derived from hypocotyl explants, cultivated in ½ MS [57] liquid medium at a photoperiod (12/12 h, light:dark). The content was elevated by the addition of ethephon (200 mg/L) at the 10th day after the inoculation in the fresh medium. The more accurate measurement of anthocyanins content, by UHPLC-PDA, revealed that the black carrot HRs derived from roots of the inbred line “43” accumulated over 4 mg/g DW of the pigments and upon the treatment with ethephon up to 8 mg/g DW. Eight anthocyanins, derivatives of cyanidin and pelargonidin, were identified and quantified using UHPLC-PDA-TOF MS [56]. Supplementation of the nutrient medium with 60 g/L sucrose, increased dry mass and anthocyanin accumulation in the culture. Elicitation with 200 µM of H_2_O_2_, on 12th day of culture resulted in about 20% higher anthocyanin content [58].

Two gene-encoding transcription factors: a bHLH gene *AmDelila* and an R2R3-MYB gene *AmRosea1* were concomitantly introduced into the HRs of common snapdragon and induced expression of anthocyanin-related genes in the roots. Further research demonstrated that *AmRosea1* alone was enough to start anthocyanin accumulation in the root tissues. The total anthocyanin content in the roots expressing *AmRosea1* reached 2 mg/g FW versus 0.3 mg/g FW in the control HRs [59].

Overexpression of PAP1 (production of anthocyanin pigment 1) gene in *Panax ginseng* C.A. Meyer (Araliaceae) HRs induced expression of phenylpropanoid and flavonoid biosynthetic pathway genes and led to 191- to 341-fold increase in anthocyanins production in comparison to the controls. The increase of anthocyanin accumulation was accompanied by the rise in the antioxidant and radical scavenging activity of *PAP1*-overexpressing roots and their improved anti-melanogenic activity [60]. Further investigation suggested improved antimicrobial and anti-elastase activities of *PAP1*-overexpressing ginseng HRs [61].

#### 2.1.2. Flavonols and Flavanols

Except for the optimization of culture conditions (light, growth regulators, sucrose concentration, bacterial strain used to initiate the culture) and overexpression of genes from the *myb* gene family, elicitation with abiotic (UV-B irradiation) and biotic (yeast extract, chitosan, inactivated fungal preparations) elicitors was used to enhance production of flavonols and flavanols in the HRs. Plant hormones (methyl jasmonate, MeJa) and plant growth regulators (ethephon), in some studies classified as abiotic elicitors [16], were also applied to stimulate biosynthesis of the compounds.

**Figure 3:**
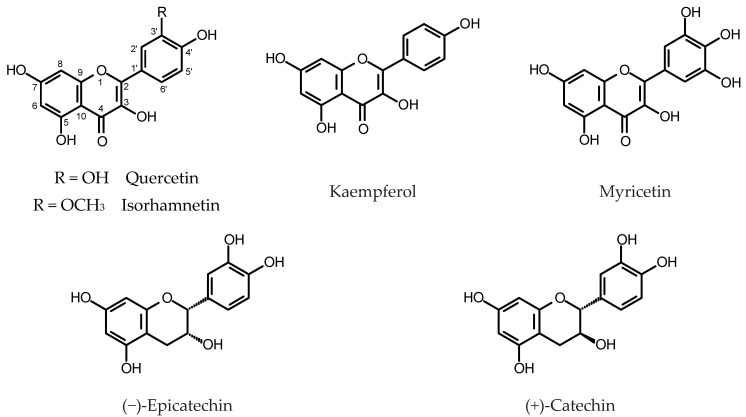
Chemical structures of selected flavonols (upper row) and flavanols.

HRs of buckwheat (*Fagopyrum esculentum* Moench) and Tartary buckwheat, except for anthocyanins and PAs, accumulated substantial amounts of flavonols (kaempferol, quercetin, rutin) and flavanols (catechin, epicatechin). Trotin et al. [62] estimated contents of (+)-catechin, (−)-epicatechin and (−)-epicatechin 3-*O*-gallate (for structures, see Figure 3) in a HR culture of buckwheat obtained by transformation with *R. rhizogenes* 15834. (−)-Epicatechin gallate was the major flavanol found in the roots. Its content reached 10 mg/g DW after 21 days of culture and was like that estimated in normal root culture. The HRs produced twice as much of (+)-catechin as normal roots (up to 8 mg/g DW) but the content of (−)-epicatechin in the transformed roots was significantly lower. It is worth noting, that after 21 days in culture dry weight of roots reached 720 mg DW per flask and 180 mg DW per flask for hairy and normal roots, respectively. The HRs of buckwheat accumulated higher yields of rutin (quercetin 3-*O*-rutinoside, 1.3 mg/g DW) than did untransformed roots (0.5 mg/g DW) [63]. Overexpression of *Arabidopsis* transcription factor *AtMYB12* in buckwheat HRs led to the increased expression levels of flavonoid biosynthetic enzymes and enhanced rutin production in comparison with the control HRs [64]. Roots of Tartary buckwheat cv. “Hokkai T8” and “Hokkai T10”, obtained by infection of hypocotyl explants with *R. rhizogenes* strain R1000 were cultivated in both light and dark conditions. The roots contained higher amounts of flavonols when exposed to light. Quercetin content reached its maximum (nearly 1 mg/g DW) 5–10 days after the transfer of roots to the fresh medium and then dropped, whereas the highest content of rutin was found after 15 days of culture (59 mg/g DW) [35]. Catechin and rutin contents in the HRs of Tartary buckwheat differed depending on the *R. rhizogenes* strain that was used to obtain the culture. The quercetin content remained unchanged irrespective of the bacterial strain used [53]. The ethephon treatment (0.5–2.0 mg/mL) significantly increased rutin accumulation in the roots [54]. A similar effect was achieved with overexpression of FtMYB6, an SG7 R2R3-MYB transcription factor induced by light [65]. Overexpression of FtUGT73BE5, an UDP-glycosyltransferase, in the HRs of Tartary buckwheat caused increase in rutin content in the biomass from 0.77 to 1.29-fold when compared with the control (up to 80 mg/g DW) [66]. Choi et al. [67] investigated expression of 11 genes of the phenylpropanoid biosynthetic pathway in seedling roots, adventitious roots and HRs of *F. tataricum*. Five of the examined genes were highly expressed in the studied HR clones. The HRs accumulated significantly higher amounts of (+)-catechin, (−)-epicatechin and rutin than those found in the untransformed axenic roots and roots of the seedlings.

Tusevski and coworkers [68], by means of HPLC-PAD-ESI-MS^n^, identified and quantified numerous phenolic compounds produced by HRs of *Hypericum perforatum* L. (Hypericaceae) obtained by a transformation with *R. rhizogenes* A4. Flavonols (quercetin 6-*C*-glucoside, unidentified isorhamnetin-*O*-hexoside, rutin, kaempferol), flavanol (catechin) and two proanthocyanidin dimers were present in the dark grown roots in small quantities (<1 mg/g DW). Contents of epicatechin and one proanthocyanidin dimer exceeded 1 mg/g DW. The phenolic profile changed when the roots were exposed to light [69]. Selected lines of *H. perforatum* HRs, transformed with *R. rhizogenes* A4M70GUS, accumulated up to 4.7 mg/g DW catechin, negligible amounts of procyanidin dimers, and up to 5.9 mg/g DW of quercetin and kaempferol derivatives [70].

Overexpression of *LcMYB1* in lychee HRs led to the twofold increase in total flavonol content, in reference to the control roots [49]. The HRs of *Linum album* Kotschy ex Boiss. (Linaceae) transformed with *R. rhizogenes* LBA9402, upon elicitation with a cell wall preparation from *Piriformospora indica* (1%, *v*/*v*; 48–120 h treatment)*,* demonstrated enhanced expression of genes-encoding several enzymes of phenylpropanoid biosynthetic pathway and enhanced flavonols and flavanols accumulation. The roots accumulated up to 0.1 mg/g DW catechin, kaempferol (<3.6 µg/mg DW) and myricetin (<2.4 µg/mg DW). The total flavonoid content did not exceed 0.15 mg/g DW [71,72,73]. According to Thiruvengadam et al. [74] HRs of *Polygonum multiflorum* Thunb. (Polygonaceae) transformed with *R. rhizogenes* strain KCTC 2703 produced rutin, myricetin, quercetin and kaempferol, although in low quantities (0.35, 0.40, 0.29 and 0.11 mg/g DW, respectively). In the HR cultures of *P. multiflorum* transformed with *R. rhizogenes* KCCM 11,879 quercetin was the most abundant flavonol (up to 4.59 mg/g DW). Elicitation with MeJa (50 µM) caused a 3.83-fold increase in quercetin content [75].

A series of papers on HR cultures of *Momordica charantia* L., *Momordica dioica* Roxb. ex Willd., *Cucumis anguria* L. (Cucurbitaceae), *Brassica rapa* L. ssp. *rapa* and *Brassica rapa* L. ssp. *pekinensis* (Brassicaceae) [76,77,78,79,80,81] described the quantification of flavonols by an UPLC method with UV detection, using a set of commercially available standards comprising: rutin, myricetin, quercetin, kaempferol and catechin. The estimated contents of flavonols were low (<0.8 mg/g DW), except for *M. charantia* HRs that produced over 1 mg/g DW of catechin.

HRs of *Isatis tinctoria* L. (Brassicaceae), obtained by inoculation with *R. rhizogenes* LBA9402 and cultivated in the optimum conditions, produced rutin (94 μg/g DW), quercetin (53 μg/g DW), kaempferol (134 μg/g DW) and isorhamnetin (84 μg/g DW). Contents of the flavonols were higher than those found in the roots of two-year-old *I. tinctoria* field-grown plants [82]. Elicitation with 150 mg/L of chitosan, for 36 h, led to a significantly higher flavonol accumulation (rutin—812 μg/g DW; quercetin—733 μg/g DW; kaempferol—285 μg/g DW; isorhamnetin—618 μg/g DW) [83]. A co-culture of the HRs with an immobilized *Aspergillus niger* caused oxidative stress in the plant tissue and triggered up-regulation of the flavonoid biosynthetic pathway. As a result, under the optimum conditions (ca.10^4^ spores/mL, 30 °C, initial pH value 7.0, 72 h treatment), higher contents of all flavonols but rutin were detected in the examined *I. tinctoria* HRs (quercetin—up to 1500 μg/g DW; isorhamnetin—up to 700 μg/g DW; kaempferol—up to 1000 μg/g DW) [84]. The roots exposed to UV-B radiation (108 kJ/m^2^) demonstrated substantial up-regulation of the chalcone synthase gene and increased accumulation of all monitored flavonols (rutin—up to 1500 μg/g DW; quercetin—up to 1700 μg/g DW; isorhamnetin—up to 2000 μg/g DW; kaempferol—up to 2200 μg/g DW) [85].

Ghimire et al. [86] found that elicitation either with yeast extract (100 mg/L) or with MeJa (100 μM) significantly increased flavonol accumulation in the HRs of *Aster scaber* (Asteraceae, full botanical name not provided). Upon elicitation with MeJa, biomass of the roots contained: 2.07 mg/g DW of myricetin, 0.69 mg/g DW of quercetin, 0.26 mg/g DW of kaempferol and 0.19 mg/g DW of rutin. The HRs of *Ligularia fischeri* Turcz. f. *spiciformis* (Nakai) (Asteraceae), obtained by inoculation with *R. rhizogenes* KCTC 2703, were found to produce myricetin (2.38 mg/g DW), quercetin (0.51 mg/g DW, kaempferol (0.12 mg/g DW) and rutin (0.14 mg/g DW) [87].

HRs may be a source of new, previously unknown natural products. Two unusual, biologically active, derivatives of kaempferol and quercetin (4′-*O*-methylkaempferol-3-*O*-[(4′′→13′′′)-2′′′,6′′′,10′′′,14′′′-tetramethylhexadecan-13′′′-olyl]-β-_D_-glucopyranoside and 3′,4′-di-O-methylquercetin-7-O-[(4′′→13′′′)-2′′′,6′′′,10′′′,14′′′-tetramethylhexadec-13′′′-ol-14′′′-enyl]-*β*-_D_-glucopyranoside) were isolated from the HRs of *Catharanthus roseus* (L.) G. Don (Apocynaceae) [88].

#### 2.1.3. Flavones and Flavanones

The procedures employed to enhance the production of flavones and flavanones in the HRs were similar to those described earlier for the other groups of flavonoids and include overexpression of the genes-encoding biosynthetic enzymes (*CHI*, *PAL*, *C4H* and *4CL*), elicitation (UV-B irradiation, MeJa, yeast extract, bacterial lysates, *β*-cyclodextrin, iron oxide nanoparticles) and heterologous expression of transcription factors.

**Figure 4:**
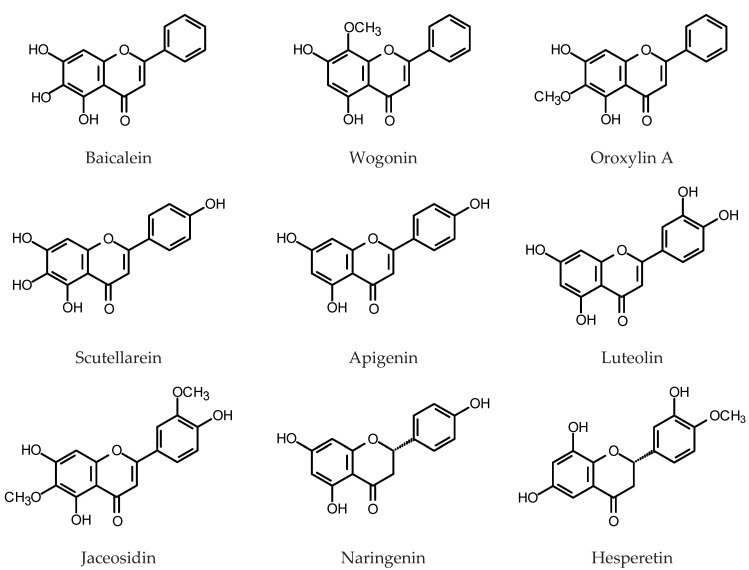
Chemical structures of selected flavones and flavanones.

One of the most spectacular examples of a plant that accumulate substantial amounts of flavones is *Scutellaria baicalensis* Georgi (Lamiaceae). Roots of the plant contain up to 27% DW of flavones, mainly glycosidic derivatives of baicalein, wogonin and oroxylin A [89] (for structures, see Figure 4). *S. baicalensis* is valued for its medicinal properties [90,91], including the antioxidant activity of its major constituents [92]. First attempts to develop a HR culture system to produce pharmacologically active flavones of *S. baicalensis* were made in the 1990s [93,94,95]. Those studies, apart from the identification of over twenty known phenolic compounds synthesized by the roots, including flavones and phenylethanoids, led to the isolation and identification of two formerly unknown flavones. However, the yields of biologically active flavones from the cultures were unsatisfactory (up to 1.2% DW of baicalein 7-*O*-glucuronide). The HRs that emerged as a result of *S. baicalensis* transformation with *R. rhizogenes* LBA 9402, in optimum culture conditions produced 6.9% DW of total baicalein glycosides and 1.5% DW of total wogonin glycosides, measured by HPLC after the acidic hydrolysis of the native compounds [96]. Kuzovkina et al. [97,98], by a transformation of *S. baicalensis* with *R. rhizogenes* A4 obtained HRs that produced 5% DW of flavonoids (spectrophotometrically measured). The content increased 1.8-fold after the 72 h of treatment with 100 μM of MeJa. More accurate quantification by HPLC revealed 1.27% DW of baicalin (baicalein 7-*O*-glucuronide), 1.07% DW of wogonoside (wogonin 7-*O*-glucuronide) and 0.38% DW of their aglycones in the analyzed roots. Maximum yields of baicalin and wogonoside after MeJa treatment reached 10 mg/g DW and 24 mg/g DW, respectively [98,99]. Hirotani et al. [100] found that accumulation of mRNA for an enzyme UDP-glucose:baicalein 7-*O*-glucosyltransferase (UBGT) in the HRs of *S. baicalensis* was induced in response to wounding and salicylic acid treatment. Overexpression of chalcone isomerase gene (*SbCHI*) in the HRs of Baikal skullcap resulted in the increased accumulation of baicalin (42–60 mg/g DW), baicalein (8.9–12.1 mg/g DW) and wogonin (2.2–5.4 mg/g DW) in reference to GUS-control HR line. As expected, *SbCHI*-silenced HRs produced less flavones than the controls [101]. The HRs overexpressing phenylalanine ammonia-lyase genes (*SbPAL1*, *SbPAL2* or *SbPAL3*) produced significantly higher amounts of flavone aglycones (baicalein: 11–29 mg/g DW; wogonin 2.5–6.7 mg/g DW) than the GUS-control HRs and wild type field-grown roots. Baicalin content in PAL-overexpressing roots (57–136 mg/g DW) was slightly lower than that in wild type field-grown roots (146 mg/g SbCYP82D2DW) but much higher than that found in the GUS-control HRs [102]. Also, overexpression of cinnamate 4-hydroxylase (C4H) and 4-coumaroyl CoA ligase (4CL) increased flavone production in *S. baicalensis* HRs [103]. Heterologous expression of the transcription factor Lc from *Zea mays* in the HRs of Baikal skullcap led to the enhanced accumulation of baicalin, baicalein and wogonin in the roots, but higher yields were achieved when the *Arabidopsis* PAP1 transcription factor was overexpressed in the same experimental system (up to 102 mg/g DW of baicalin) [104]. Some improvement in yields of flavones from the cultures was achieved as well by an optimization of the nutrient medium composition [96,105,106]. Aglycones:glucuronides ratios in the cultures may be at least partially controlled by the activities of the endogenous β-glucuronidase of Baikal skullcap (baicalinase, sGUS) and baicalein 7-*O*-glucuronosyltransferase (UBGAT) [107,108]. Hydroxylation of chrysin is the step in the biosynthesis of baicalein and wogonin that requires the respective CYP450 enzymes: flavone 6-hydroxylase (F6H) and flavone 8-hydroxylase (F8H). Two enzymes: SbCYP82D1.1 (F6H) and SbCYP82D2 (F8H) from *S. baicalensis* were described and the role of *SbCYP82D2* in the wogonin biosynthesis was confirmed by its silencing in the HRs of the plant [109]. Baikal skullcap collected in the Dauria region (shared by Mongolia, Russian Federation and China) demonstrated high contents of polymethoxylated flavones. The HRs obtained from the plants retained their metabolic profile [110].

Several other species of the *Scutellaria* genus like *S. lateriflora* L., *S. andrachnoides* Vved., *S. bornmuelleri* Hausskn. ex Bornm. *S. przewalskii* Juz. and *S. pycnoclada* Juz. Bunge were also investigated in respect of their prospective use as a source of biologically active flavones [111,112,113,114,115,116,117]. American skullcap (*S. lateriflora*) is the second most popular species of *Scutellaria* used for medicinal purposes. Wilczańska-Barska et al. [111] obtained HRs of *S. lateriflora* by a transformation with *R. rhizogenes* strain A4. The roots produced a phenylethanoid glycoside—acteoside (19 mg/g DW) and flavones (scutellarin 0.6 mg/g; baicalin 14.5 mg/g; wogonoside 12.0 mg/g; wogonin 11.5 mg/g; chrysin 0.1 mg/g DW). Except for the wogonin, flavone contents were lower than those in *S. baicalensis* HRs [111,112]. The root culture of *S. lateriflora* upon elicitation with yeast extract (YE, 200 mg/L) demonstrated increased biomass accumulation and enhanced acteoside production. The optimum for flavones accumulation was 50 mg/L YE (flavone total content ca. 50 mg/g DW). Bacterial lysates applied as elicitors were less effective [111]. The HRs of the American skullcap transformed with *R. rhizogenes* ATCC 15834 were cultivated under continuous illumination or in the dark and elicited with *β*-cyclodextrin and MeJa [113]. Maximum contents of baicalein and wogonin (5.4 mg/g DW and 0.71 mg/g DW, respectively) were found in the cultures maintained in the dark, treated with 15 mM of *β*-cyclodextrin. Maximum contents of flavone glycosides—scutellarin (scutellarein 7-*O*-glucuronide) and wogonoside (ca. 0.5 mg/g DW each) were accumulated in the roots grown in the light using the same treatment. *β*-Cyclodextrin (15 mM) turned out to be more effective than MeJa (100 µM) as an inducer of flavone accumulation, after 24 h treatment. Better yields of the flavones (baicalin—22.5 mg/g DW; wogonin—5.4 mg/g DW) were achieved by Tuan et al. [114] after 72–96 h treatment with 100 µM MeJa. The HRs used in the experiment were obtained by the transformation with *R. rhizogenes* strain R1000. Stepanova et al. [115] investigated HRs of *S. baicalensis*, *S. lateriflora*, *S. przewalskii* and *S. pycnoclada* derived from the transformation of the plant material with wild-type *R. rhizogenes* A4. The roots of *S. baicalensis* and *S. przewalskii* showed the best growth indices when cultured in a liquid nutrient medium. Baicalin was the major flavone accumulated in all examined cultures. The HRs of *S. przewalskii* were characterized by a high content of flavones (ca. 33 mg/g DW in total) compared to the HRs of *S. baicalensis* (ca. 17 mg/g DW) and HRs of the two remaining species (about 5–13 mg/g DW). The HRs of *S. andrachnoides* and *S. bornmuelleri* produced low amounts of flavones [116,117]. The HRs of *S. viscidula* Bunge, *S. orientalis* L. and *S. araxensis* Grossh. have not been examined in respect of their flavone content yet [118,119].

*Dracocephalum kotschyi* Boiss (Lamiaceae) is a rare plant of medicinal properties that synthesizes polymethoxylated flavones and flavonols of antioxidative and anti-inflammatory activity [120]. The HRs of the plant induced by inoculation with *R. rhizogenes* LBA 9402 produced: apigenin, cirsimaritin, isokaempferid, penduletin, xanthomicrol and calycopterin, but the contents of individual compounds varied widely depending on the root clone. The HRs were a better source of flavonoids than the roots of the intact plant. The leaves of *D. kotchyi*, however, contained nearly eight times more flavonoids (1.7 mg/g DW) than the most productive HR clone [121]. A HR culture of *D. kotschyi* transformed with *R. rhizogenes* ATCC 15834 accumulated 0.19 mg/g FW of apigenin. Upon elicitation with iron oxide nanoparticles (75 mg/L, 24 h) the apigenin content increased up to 0.37 mg/g FW [122].

The genus *Saussurea* comprises ca. 400 plant species. Some of the plants from the genus have been traditionally used as medicines [123,124]. The HRs of *S. medusa* Maxim, transformed with *R. rhizogenes* strain R1601 and maintained in the liquid N6 medium [125], produced ca. 6.1 mg/g DW jaceosidin [126] in a 24–28-day culture. Overexpression of chalcone isomerase gene from *S. medusa* in the HRs of *S. involucrata* Kar. et Kir. ex Maxim., led to the increased accumulation of apigenin (ca. 2.6 mg/g DW) and a higher content of total flavonoids [127].

*Erigeron breviscapus* (Vaniot) Hand.-Mazz. (Asteraceae) synthesizes pharmacologically active flavones apigenin and scutellarein together with their glycosides. The major flavone accumulated by the plant is scutellarein. The HRs of *E. breviscapus* were obtained by transformation with *R. tumefaciens* strain C58C1 harboring pRiA4 plasmid or with the same bacterial strain modified by introducing a construct containing *EbCHI* gene encoding chalcone isomerase. The roots overexpressing EbCHI produced 2.2 mg/g DW of scutellarin, whereas the roots of the intact plant contained 0.21 mg/g DW of the compound (whole plant 2.6 mg/g DW). Elicitation with MeJa induced expression of several genes engaged in the flavonoid biosynthesis, including the genes-encoding chalcone synthase (CHS) and chalcone isomerase (CHI). The HRs of *E. breviscapus* treated with MeJa transiently accumulated up to 4.7 mg/g scutellarin [128].

HRs of *Catharanthus roseus* (L.) G. Don (Apocynaceae) harboring the gene encoding tryptophan feedback-resistant anthranilate synthase holoenzyme (*ASαβ*) produced naringin (naringenin 7-*O*-neohesperidoside) as the major phenolic metabolite (0.65–0.86 mg/g DW). Hesperidin (hesperetin 7-*O*-rutinoside) was accumulated in the roots in smaller amounts (0.11–0.23 mg/g DW) [129].

Silencing of two flavone synthase II (FNSII) genes: *GmFNSII 1* and *GmFNSII -2*, in the HRs of soybean cultivar Hefeng 47, substantially decreased apigenin accumulation and reduced tolerance to salt stress but increased isoflavonoid production in the investigated root clones [130]. Glucose, mannitol, MeJa and NaCl significantly increased expression of *GmFNSII 1* and *GmFNSII -2* in the HRs of soybean. The motifs responsive to MeJa and glucose were found in the *GmFNSII 1* and *GmFNSII -2* promoter sequences. It was suggested that the oxidative damage induced by the salt stress may be mitigated by the flavone accumulation [131].

Components of the flavonoid fraction were analyzed in roots, HRs and a cell suspension culture derived from roots of *M. truncatula* [132]. *M. truncatula* HRs were obtained by inoculation with *R. rhizogenes* strain Arqua1. The HRs contained three luteolin glycosides, chrysoeriol and two flavanones: naringenin and liquiritigenin. Extracts from roots of the intact plant analyzed by the same method revealed the presence of seven flavone glycosides, derivatives of luteolin and chrysoeriol. Flavonoid aglycones, as well as flavanones, were not detected in the roots of the plant. Five flavones: orientin (luteolin 8-C-glucoside), vitexin (apigenin 8-C-glucoside), isovitexin (apigenin 6-C-glucoside), luteolin and apigenin were quantified in seeds, leaves, roots, calli and HRs of *Cajanus cajan* (L.) Millsp. (Fabaceae). The contents of orientin, vitexin and isovitexin in the HRs were higher than those in the roots of the intact plant but much lower than those detected in the plant leaves. Apigenin and luteolin contents in the roots of pigeon pea plants were significantly higher than those in the HRs [133]. An effect of UV-B irradiation on accumulation of phenolic constituents in *C. cajan* HRs was investigated by Gai et al. [134]. Contents of orientin, vitexin, isovitexin, luteolin and apigenin increased after 2–8 h UV-B treatment and were 1.27–4.44-fold higher than those in the control roots. However, the yields of the individual compounds were low. The content of the major flavone, luteolin, after 2 h of UV-B irradiation reached 19 µg/g DW.

#### 2.1.4. Isoflavonoids

Isoflavonoids, like the remaining flavonoids, are regarded as dietary antioxidants, i.e., compounds that may protect against oxidative stress linked to inflammation and the risk of macromolecule damage by free radicals. The group of compounds includes isoflavones, isoflavanones, isoflavans, rotenoids and pterocarpans. Although they are reported from many plant families, isoflavonoids are particularly abundant in Leguminosae plants. The most known dietary source of the compounds are the yellow-skin seeds of *Glycine max* [135,136]. While the isoflavones are normally present in relatively low amounts in mature soybean tissues, several attempts were made to develop soybeans that accumulate much higher contents of isoflavones than those found in the wild-type seed.

**Figure 5:**
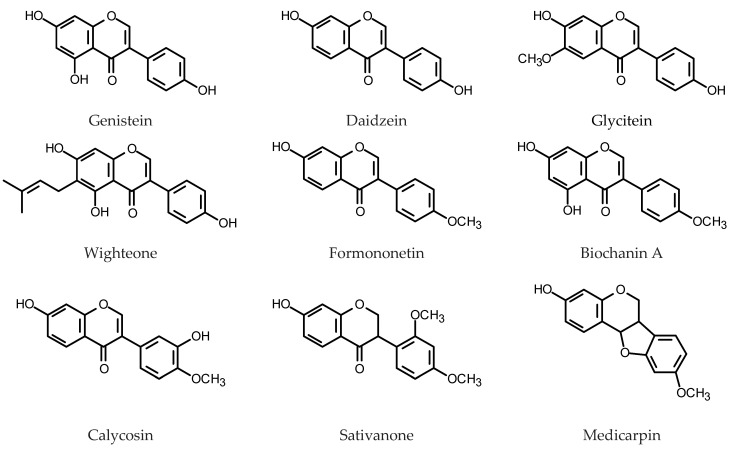
Chemical structures of selected isoflavonoids.

To study the role of isoflavonoids in the plant resistance to fungal infection, HRs were initiated from two soybean genotypes with different susceptibility to the SDS disease (sudden death syndrome) caused by the soil-borne fungal pathogen, *Fusarium solani* f. sp. *glycines*. Daidzein derivatives predominated in the isoflavone fraction extracted both from the soybean hairy roots and intact soybean. The principal isoflavones (genistin, daidzin, their malonyl conjugates and aglycones) and isoflavonoid phytoalexins (coumestrol, coumestrol conjugates and glyceollin) (for structures, see Figure 5) were determined in extracts from the *Fusarium*-inoculated and non-inoculated hairy roots. Inoculation with *F. solani* negatively affected accumulation of all monitored isoflavonoids except for glyceollin. The compound demonstrated better antifungal activity against the investigated *Fusarium* species than the remaining isoflavonoids used in the assay (daidzin, daidzein, genistin, genistein, glycitin, glycitein) [137].

Simultaneous silencing of flavone synthase II genes (*GmFNSII-1* and *GmFSNII-2*) in the HRs of soybean led to the reduction or cessation of apigenin biosynthesis. At the same time, increased accumulation of genistein was observed [130]. Synchronous silencing of *FNSII* and flavanone-3-hydroxylase (*F3H*) genes caused an increase of daidzein content in soybean HRs up to 1.55 mg/g DW (0.9 mg/g DW in control roots) [138]. Overexpression of *GmMYB100*, a gene encoding a R2R3 MYB transcription factor, in *G. max* HRs resulted in diminished expression of the genes-encoding enzymes engaged in flavonoid biosynthesis and diminished isoflavone content. Silencing of the *GmMYB100* did not affect expression of the genes for the biosynthetic enzymes (*GmCHS7, GmCHS8, GmCHI, GmIFS, GmF3H*) but significantly increased isoflavonoid content [139]. Soybean cotyledon HRs harboring *GmIFS1* showed better tolerance to salt stress and increased isoflavone content under salt stress conditions [140]. GmMYB58 and GmMYB205 are seed-specific flavonoid biosynthesis activators. Their overexpression in soybean HRs increased transcription of: *GmCHS8*, *GmCHI*, *GmIFS2*, *GmFLS1*, *UGT73F2* (isoflavone UDP-glucosyltransferase gene) and *IF7Mat* (isoflavone 7-*O*-glucoside-6”-*O*-malonyltransferase gene). An unidentified daidzein derivative, which dominated the isoflavonoid profile of the control roots (1 mg/g DW), was excessively produced by the transgenic HRs. The daidzein derivative content reached 3.2 mg/g DW in *GmMYB58*-overexpressing HRs and 6.7 mg/g DW in the roots overexpressing *GmMYB205* [141]. Soybean isoflavonoids are mainly glycoconjugates and their biosynthesis is catalyzed by different UDP-glycosyltransferases (UGT). Six genes for GmUGTs were overexpressed in soybean HRs. In the transgenic root lines overexpressing UGT72Z3, UGT73C20 and UGT88E19, the total content of isoflavonoids increased 1.1- to 1.6-fold in reference to the control and reached over 7 mg/g DW [142]. Increased resistance to *Phytophthora sojae* infection was observed in *G. max* HRs overexpressing *GmCHI1A* (chalcone isomerase gene from the soybean cv. Nannong 10-1). This observation agreed with experimentally proven induction of *GmCHI1A* expression and enhancement of daidzein accumulation by *P. sojae* [143]. Fungal infection in soybean seedlings enhance also glycitein production. The last step in the biosynthesis of this compound is methylation catalyzed by isoflavone *O*-methyltransferase (IOMT). *IOMT*, when co-expressed with the flavonoid 6-hydroxylase gene (*F6H*, normally not expressed in the HRs) increased the content of glycitein-related metabolites by ca. 100% compared to the control [144]. The HRs overexpressing *GmMaT2* and *GmMaT4*, genes-encoding malonyl-CoA:flavonoid acyltransferases, produced more malonyldaidzin, malonylgenistin and malonylglucitin than the control. The major isoflavone, malonyldaidzin, content reached ca. 6.3 mg/g FW in *GmMaT2* overexpressing HRs. *GmMaT2* but not *GmMaT4* knockdown resulted in the reduced accumulation of malonylated isoflavonoid glycosides and reduced nodule numbers. *GmMaT2* is also upregulated by the rhizobial infection what implicates its participation in the nodulation process and malonylated isoflavone secretion into the rhizosphere [145]. An attempt was made to enhance isoflavonoid production in soybean HRs by elicitation with MeJa (100 μM), salicylic acid (SA, 200 μM), sonication and vacuum infiltration. Treatment with MeJa (72 h) resulted in 10.67-fold higher total isoflavonoid content (53.16 mg/g DW) than that in the untreated control. Daidzin content reached ca. 33.9 mg/g DW. Elicitation with SA, after 96 h, caused 5.78- and 65-fold increase in total isoflavonoid and genistein contents, respectively. Maximum isoflavonoid production, 75.26 mg/g DW, was achieved with 2 min sonication and subsequent 2 min vacuum infiltration of HRs on 30th day of the culture [146].

Pigeon pea HRs produced genistin (0.11 mg/g DW) and genistein (0.04 mg/g DW) [133]. Their accumulation in the HRs was enhanced by an exposition to UV-B radiation [134]. Leaves of *Lotus japonicus* cv. Miyakojima line MG-20 seedlings supplied with genistein produced isoprenylated isoflavonoid, wighteone. This observation led to the identification of novel prenyltransferase gene *LjG6DT.* The enzyme encoded by the gene worked only with genistein as a substrate and its expression was induced by the reduced glutathione (GSH), MeJa and SA. Overexpression of *LjG6DT* in *L. japonicus* HRs, together with GSH and genistein supplementation, induced enhanced wighteone production (0.18–0.24 µg/g DW) [147]. Twenty isoflavonoids, derivatives of 2ʹ-hydroxyformononetin, afrormosin, biochanin A, daidzein, formononetin, genistein and irisolidone were detected in the HRs of *M. truncatula*. In the roots of the intact plant 32 isoflavonoids were detected and tentatively identified. In contrast to the plant roots HRs preferably accumulated isoflavonoid aglycones [132].

Red clover (*Trifolium pratense* L., Fabaceae) is a forage legume producing formononetin, biochanin A, daidzein and genistein as major isoflavone constituents [148]. Kumar et al. [149] described stable production of isoflavones by HRs of *T. pratense*. One of the fast-growing HRs clones, displayed a high accumulation of all four pharmaceutically important isoflavones: daidzein (8.56 mg/g DW), genistein (2.45 mg/g DW), formononetin (15.23 mg/g DW) and biochanin A (1.10 mg/g DW). The HRs of *T. pratense* var. URS-BRS Mesclador were obtained by inoculation with *R. rhizogenes* strain A4TC24 and 23 isoflavonoids were tentatively identified as their metabolites. The isoflavonoids were, putatively, derivatives of: daidzein, pseudobaptigenin, pratensin, biochanin, irilone, formononetin and 3ʹ,7-di-*O*-methylorobol. Isoflavonoid contents were monitored in the selected clones of roots up to five months of culture. One of the examined clones (6HR) accumulated 5.5 mg/g DW biochanin A, 20.8 mg/g DW formononetin and ca. 0.7 mg/g genistein after 90 days of culture. The HRs were elicited, 7 days after subculture, using either elevated sucrose content in the medium (60 g/L) or SA (10 and 30 mg/L). Sucrose (60 g/L) increased accumulation of isoflavonoids in the HRs. After 3.5 days of treatment, roots of clone 4HR contained 12.8 mg/g DW daidzein, 0.7 mg/g DW genistein, 13.4 mg/g DW formononetin and 1.1 mg/g DW biochanin A, whereas the roots of clone 8HR produced 1.9 mg/g DW daidzein, 0.4 mg/g DW genistein, 14.8 mg/g DW formononetin and 4.1 mg/g DW biochanin A. Untreated roots (controls) accumulated 1.2–2.9 mg/g DW daidzein, 0.1–1.3 mg/g DW genistein, 2.1–3.0 mg/g DW formononetin and 0.7–4.4 mg/g DW biochanin A. Elicitation with SA was less effective than the treatment with sucrose [150].

*Astragalus membranaceus* (Fisch.) Bunge (proper name *A. propinquus* Schischkin, Fabaceae) has a long history of use in traditional Chinese medicine. Roots of the plant are also commercially available in Europe and USA as dietary supplements and functional foodstuffs. Calycosin and calycosin 7-*O*-glucoside were the major isoflavone components of the HRs derived from the plant. The two compounds, together with ononin (formononetin 7-*O*-glucoside), formononetin and astraisoflavan 7-*O*-glucoside were quali-quantitatively determined by LC-MS/MS. Under optimum conditions, the total isoflavonoid content in 34-day old *A. membranaceus* HRs reached 0.24 mg/g DW. This yield was significantly higher compared to that of three-year-old field-grown roots (0.19 mg/g DW) [151,152]. The HRs exposed to UV-B (86.4 kJ/m^2^) synthesized more isoflavonoids (up to 0.53 mg/g DW). All investigated genes involved in isoflavonoid biosynthesis were also up-regulated following the UV-B irradiation. *PAL* and *C4H* were found to be the key genes implicated in the control of the process [153]. Elicitation of 34 days old HRs of *A. membranaceus* with MeJa (283 µM, 33.75 h treatment) raised the content of isoflavonoids to 2.25 mg/g DW and upregulated the genes related to isoflavone biosynthesis [154]. A co-culture of *A. membranaceus* HRs with immobilized *Aspergillus niger* enhanced accumulation of calycosin (0.73 mg/g DW) and formononetin (1.12 mg/g DW) in the roots [155].

*Psoralea corylifolia* L. (Babchi, Fabaceae) is a plant used in traditional medicine of China and India. The plant accumulates coumarins, flavonoids and terpenophenols, including pharmacologically active bakuchiol, which gained some popularity as a component of cosmetics [156,157]. The first study on isoflavonoid production in the HRs of *Psoralea* spp. was published in 1999 [158]. HR lines derived from seven distinct species were examined in respect of their daidzein content. The examination revealed that *P. leucantha* F. Muell. and *P. lachnostachys* F. Muell HRs were the best isoflavonoid producers. One, highly productive line (daidzein 10.2 mg/g DW, coumestrol 0.48 mg/g DW) was chosen for further investigation. The roots were elicited with 30 mg/L of chitosan at the end of the exponential phase of growth (21st day). After the addition of chitosan, the content of daidzein in biomass dropped to ca. 8 mg/g DW due to the release of the compound into the culture medium. Coumestrol and genistein contents in the roots increased from ca. 0.40 to 0.52 mg/g DW and from ca. 0.03 to 0.07 mg/g DW, respectively. Coumestrol was also liberated to the medium after the elicitation with chitosan. HR cultures of *P. corylifolia* L., transformed with *R. rhizogenes* ATCC 15834, similarly to the control roots, accumulated daidzin as a major isoflavonoide. The compound was accompanied with smaller amounts of formononetin glucoside, genistin and daidzein. Bakuchiol was absent from the cultures [159]. Clones of *P. corylifolia* HRs, obtained by inoculation with *R. rhizogenes* LBA 9402, in optimum conditions produced up to 20.6 mg/g DW daidzein and up to 3.7 mg/g DW genistein [160].

*Pueraria* spp. (Fabaceae) due to isoflavonoid content is traditionally used to relieve menopausal symptoms [161]. The HRs of *Pueraria phaseoloides* (Roxb.) Benth induced by *R. rhizogenes* ATCC 15834 contained ca. 1 mg/g DW puerarin (daidzein 8-*C*-glucoside) [162]. The culture was scaled up to a 2.5 L bioreactor and the culture medium was modified to achieve better yield. In optimum conditions, roots cultivated in the bioreactor produced ca. 5.6 mg/g DW of puerarin and the product was partially liberated to the nutrient medium [163]. A HR culture of *P. candollei* Wall. Ex Benth. was established using *R. rhizogenes* ATCC 15834. The total isoflavonoid content in the roots reached 36.48 mg/g DW. Daidzin was the major product (29.9 mg/g DW), and the puerarin content was estimated as 3.4 mg/g DW [164]. Chitosan (50, 100 and 150 mg/L), MeJa (50, 100 and 200 µM), SA (50,100 and 200 µM), YE (0.5, 1 and 2 mg/mL) and autoclaved *Rhizobium* culture (1, 2 and 3% *v*/*v*) were applied as elicitors to enhance isoflavonoid production in the HRs. Though all of the applied treatments increased isoflavonoid accumulation, the yeast extract addition (0.5 mg/mL, 3 days) proved to be the most effective (60.5 mg/g DW total isoflavonoids) [165]. *P. candollei* var. *myrifica* (Airy Shaw & Suvat.) Niyomdham, except for the isoflavonoids, synthesizes another phytoestrogen, deoxymiroestrol. The HRs of the plant produced up to 7 mg/g DW isoflavonoids (mainly daidzin and genistin) and up to 77 µg/g DW of deoxymiroestrol. The contents were higher than those found in the roots of the intact plant (3.5 mg/g DW and 15 µg/g DW, respectively). Chitosan, YE and MeJa were applied to study effects of elicitation on the active metabolite content. The HRs elicited with MeJa (200 µM) accumulated puerarin (0.54 mg/g DW), daidzin (8.68 mg/g DW), genistin (5.27 mg/g DW), daidzein (0.45 mg/g DW), genistein (0.16 mg/g DW), kwakhurin (1.39 mg/g DW) and deoxymiroestrol (0.25 mg/g DW), after 6 days of the treatment. The remaining elicitors also induced productivity of the roots, though to a lesser extent [166]. *P. candollei* Grah. ex. Benth. var. *candollei* was genetically transformed using two different *R. rhizogenes* strains (ATCC 15834 and 43,057) to obtan several clones of HRs. 

The effect of the inoculum size (1 and 2% *w/v*) and temperature (25 or 32 °C) on the growth of the obtained HRs and on their isoflavonoid content was investigated. The maximum flavonoid accumulation (31 mg/g DW with daidzein and puerarin as major compounds) was found in the cultures started with 1% inoculum and cultivated at 32 °C. Moreover, it was found that cultivation of the HRs at higher temperature reduced browning of the tissue [167]. Kim et al. [168] obtained HRs from two different lines of *P. lobata* (Willd.) Ohwi (kudzu), collected in two different regions of Korea. The roots accumulated more puerarin and daidzin than the respective callus cultures, but the contents of the compounds in the tubers of the intact plants were higher. *P. lobata* C-glycosyltransferase (PlUGT43) uses daidzein and genistein as substrates. The overexpression of *PlUGT43* in soybean hairy roots that synthesize daidzein, but not puerarin, led to the production of puerarin in the transgenic roots [169].

The transformed root cultures of *Ononis spinosa* L. and *Ononis arvensis* L. (Fabaceae) were obtained by Nóra Gampe et al. [170]. The most abundant compounds in the HRs were medicarpin, sativanone and pseudobaptigenin glucosides (16.9–28.9 mg/g DW, 1.3–11.4 mg/g DW and 0.9–2.0 mg/g DW, respectively). Formononetin and onogenin derivatives were present in smaller amounts. Two new phenolic compounds were found in the HRs (bulatlactone 2”-*O*-glucoside and ononilactone). The total isoflavonoid production in the cultures was comparable to that in the wild-grown *O. arvensis* and approximately twofold higher than that in wild-grown *O. spinosa* samples.

#### 2.1.5. Miscellaneous Flavonoids

**Figure 6:**
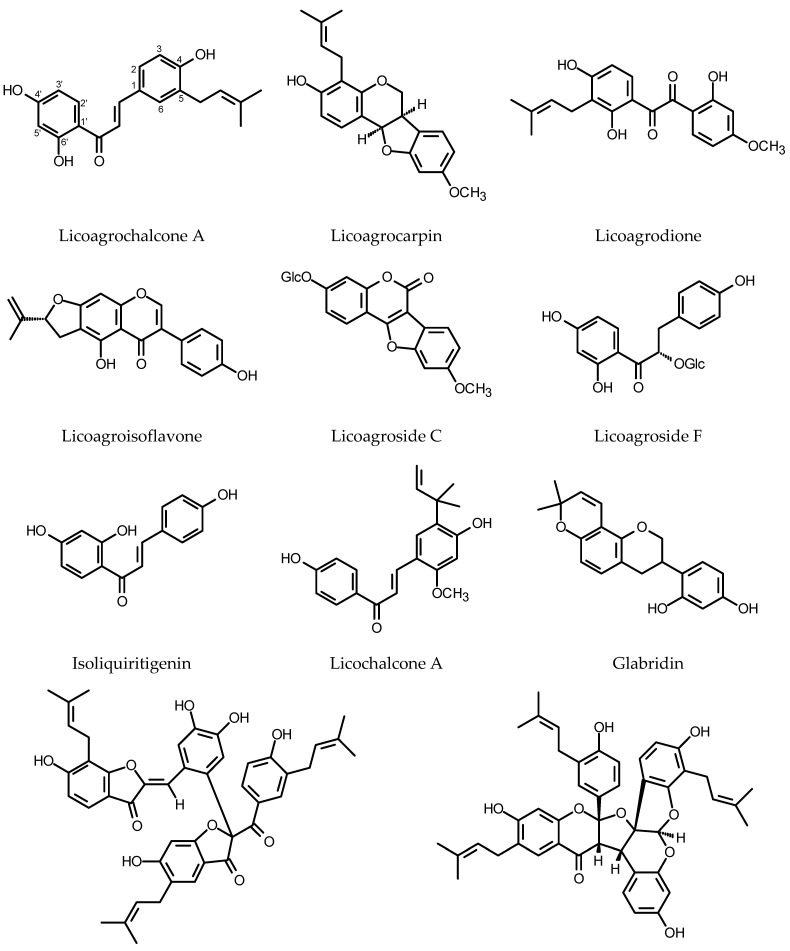

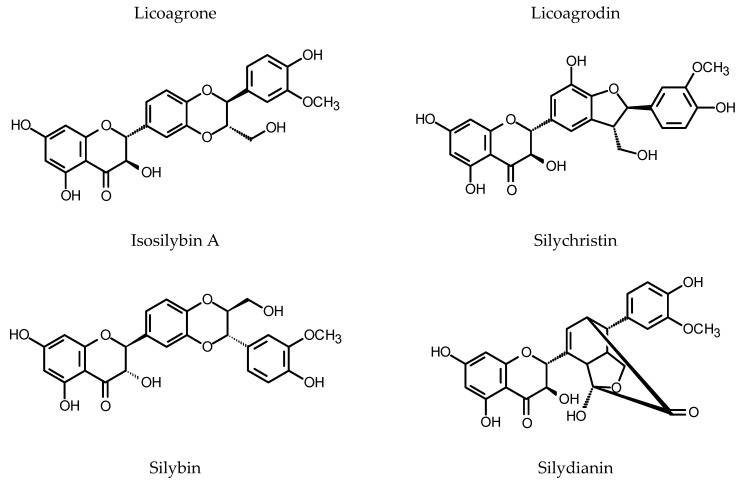
Chemical structures of selected rare and atypical flavonoids found in HRs.

Though the HRs of *Glycyrrhiza glabra* L. (Fabaceae) do not produce glycyrrhizic acid, a sweet-tasting saponin responsible for the major pharmacological activity of the liquorice root (peptic ulcer healing), they synthesize vast array of biologically active polyphenols of unique structures [171,172] (see Figure 6). Two new compounds, licoagrochalcone and licoagrocarpin (chalcone- and pterocarpan-type compounds), were isolated from the HRs of licorice transformed with a *Rhizobacterium* strain harboring pRi15834 and pBI121 (GUS). Eight known compounds were also found in the HRs, including three chalcones, four prenylated flavanones and one prenylated flavanol [173]. Further investigation of the same plant material led to the isolation of licoagrodione, another new compound, together with five known flavonoids. The ntimicrobial activities of the isolated compounds were assessed by the disc diffusion method. Glyinflanin K was the only compound that did not show activity against the bacteria and fungi used in the experiment [174]. The continuation of this research allowed for the isolation of unusual prenylated biaurone, licoagrone, together with five known flavonoids (kanzonol D, afrormosin, odoratin, phaseol, echinatin) [175]. Li et al. [176] isolated and described a new biflavonoid, licoagrodin and another four new flavonoids (licoagrochalcone B, licoagrochalcone C, licoagrochalcone D and licoagroaurone), as well as four known flavonoids. More polar fractions separated from the HRs gave licoagroside A, ononin, calycosin 7-*O*-glucoside, wistin, vicenin-2, afrormosin 7-*O*-(6”-malonylglucoside) and isoschaftoside. Licoagroisoflavon and licoagrosides C-F were isolated as new compounds from the HRs of *G. pallidiflora* Maxim. The compounds were accompanied by eleven known flavonoids [177,178]. In the HRs of *G. uralensis* Fisch. two-fold increase in total flavonoid content (up to 30 mg/g DW) was observed following the combined 48 h of treatment with YE (0.1%) and polyethylene glycol (PEG8000, 2%) [179]. Tween 80, added to 20-day-old HRs of *G. uralensis*, caused enhanced production of licochalcone A accompanied by elevated mRNA levels for PAL, 4CL (4-coumarate:coenzyme A ligase) and C4H. After 15 days of the treatment, the roots yielded over 3 mg of licochalcone A per flask, and the product was almost entirely liberated to the culture medium. The control HRs accumulated up to 0.35 mg/flask of the compound in their biomass [180]. The verexpression of *CHS* in *G. uralensis* HRs resulted in the increased production of chalcones and liquiritigenin in the transgenic roots. The number of *CHS* copies in the examined clones of HRs was determined as 9, 10, 11, 13 and 18. The root clone with nine copies of *CHS* accumulated the highest contents of flavonoids [181].

The *Genista tinctoria* L. HRs synthesized neither isoflavone, derivatives of daidzein and genistein, nor the derivatives of apigenin and luteolin characteristic of the intact plant. Instead, the roots accumulated isoliquiritigenin (daidzein precursor, 23 mg/g DW) absent from the roots of the intact plant. Abscisic acid (ABA, 37.8 μM), added to the culture on the 42nd day of growth, induced the release of the product (80%) to the culture medium [182].

Alikaridis et al. [183] investigated roots obtained by a transformation of *Silybum marianum* Gaertner with *R. rhizogenes* ATCC 15834. The roots contained only minute amounts (0.4 μg/g DW) of silychristin and silydianin. Silybins A and B were not detected, whereas the contents of isosilybins A and B were higher than those in the untransformed roots (isosilybin A—0.01 mg/g DW). The *S. marianum* HRs examined by Rahnama et al. [184] contained up to 0.14 mg/g DW silychristin. The other flavonolignans were present in smaller amounts.

### 2.2. Stilbenoids

Stilbenoids are a class of polyphenolic plant constituents that have the general structural formula C6-C2-C6 and share an initial part of their biosynthetic pathway with flavonoids. The compounds could be found in various plant species, including peanut (Fabaceae), grapevine (Vitaceae), berries (Ericaceae), pine (Pinaceae) and tomato (Solanaceae). Their main function is the protection of the host plant against pathogen infestation and oxidative stress generated by different environmental stimuli [185]. Stilbenoids, due to their biological activity, may find application as protective agents in cardiovascular disease, diabetes, neurodegeneration, obesity and other ailments [186].

The compounds were primarily detected in elicited HRs and the procedures used to enhance the productivity of the cultured roots included elicitation (MeJa, paraqat, H_2_O_2_) with concomitant use of permeabilizing agent (methyl-*β*-cyclodextrin) to liberate the products into the culture medium. R2R3-MYB-type transcription factors are engaged in the up-regulation of stilbenoid biosynthesis, and this may find an application in genetic engineering.

**Figure 7:**
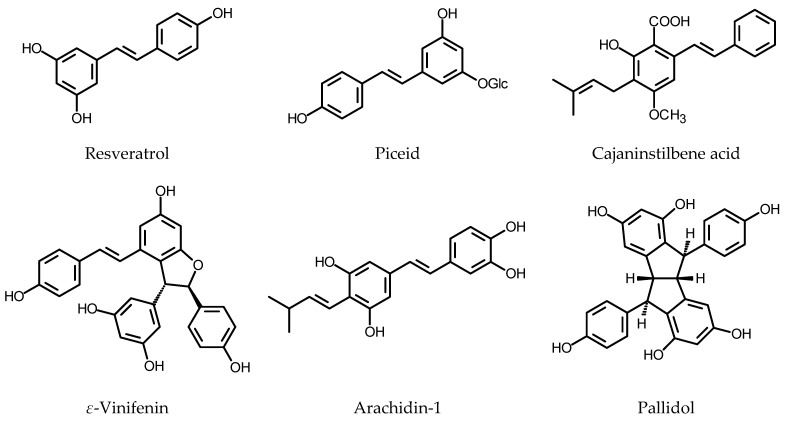
Chemical structures of selected stilbenoids produced by HRs.

Medina-Boliwar et al. [187] found that HRs obtained by the transformation of *Arachis hypogaea* L. cv. Andru II (Fabaceae) with *R. rhizogenes* ATCC 15834, upon elicitation with sodium acetate, produced stilbenoids that are excreted into the culture medium. *Trans*-resveratrol and *trans*-pterostilbene (for structures, see Figure 7) were identified as metabolites present in the elicited cultures. *Trans*-resveratrol, *trans*-arachidin-1 and *trans*-arachidin-3 were isolated from the HRs of peanut cv. Hull elicited with 10.2 mM sodium acetate. Among the three compounds, arachidin-1 was the most active as an inhibitor of lipoprotein oxidation. In the applied assay, arachidin-1 was more active than butylohydroxytoluen (BHT). In contrast to resveratrol, arachidins at a dose of 55 µM showed some cytotoxicity towards HeLa and RAW 264.7 cells [188]. The same culture was used to investigate the effects of medium optimization, and the age of the elicited roots on growth performance and root culture phenotype. The effect of elicitation on biosynthesis and liberation of stilbenoids into the culture medium was also studied. The HRs elicited on the ninth day of the culture produced *trans*-resveratrol (ca. 90 µg/g DW), *trans*-arachidin-1 (ca. 33 µg/g DW), and *trans*-arachidin-3 (ca. 37 µg/g DW) when cultivated in modified MS instead of Gamborg’s B5 nutrient medium. As the stilbenoids were secreted into the medium, the biomass of HRs contained only minute amounts of the compounds (ca. 1 µg/g DW, ca. 0.75 µg/g DW and ca. 0.71 µg/g DW, respectively) [189]. In a search for the optimum elicitation method, hydrogen peroxide (10 mM), MeJa (100 µM), methyl-*β*-cyclodextrin (9 g/L), and combination of MeJa with methyl-*β*-cyclodextrin were used as elicitors, instead of sodium acetate. The application of either H_2_O_2_ or cyclodextrin caused secretion of piceatannol, another stilbenoid formerly not found in the HRs of *A. hypogaea* cv. Hull. The contents of stilbenoids in the culture medium were monitored until 96 h after elicitation. Individual compounds reached their maximum content in the analyzed medium at different time intervals. The best results were achieved with HRs treated with the combination of MeJa and cyclodextrin. Sixty hours after elicitation, the roots produced resveratrol (ca. 5.3 mg/g DW), piceatannol (ca. 0.3 mg/g DW), arachidin-1 (ca. 4 mg/g DW) and arachidin-3 (ca. 17.1 mg/g DW) [190].

Yang et al. [191] found that the prenyl subunit of the prenylated stilbenoids like arachidins originated from the plastidic terpenoid pathway. Moreover, they purified and described membrane-bound stilbenoid-specific prenyltransferase from peanut HRs. Elicitor-treated *A. hypogaea* HRs were used to discover genes-encoding stilbenoid prenyltransferases. Transcripts encoding five enzymes were identified and two of the enzymes were characterized, including AhR4DT-1 that catalyzes prenylation of resveratrol at C-4 to form arachidin-2 [192]. The prenylated stilbenoids of the peanut manifest an array of interesting biological activities (e.g., anti-inflammatory, antiviral, antioxidant and cytotoxic towards human cancer cells) and have better bioavailability than resveratrol [193,194,195]. To achieve higher yields of the prenylated stilbenoids from peanut HRs, the elicitation procedure was further optimized using 125 µM MeJa (signaling molecule implicated in secondary metabolism regulation), 18 g/L cyclodextrin (permeabilizing agent that may trap the product and prevent feedback inhibition), 3 mM H_2_O_2_ (inducer of piceatannol production in the HRs of the peanut) and 1 mM MgCl_2_ (Mg^2+^ as a co-factor of resveratrol prenyltransferases) [194]. The formerly applied treatment [190] allowed for ca. 56 mg/L of arachidin-1 and ca. 148 mg/L of arachidin-3 to be obtained. With the new procedure, the yields increased to 227.4 mg/L and 370.6 mg/L, respectively. Moreover, arachidin-2 (83.1 mg/L) and arachidin-5 (68.4 mg/L) were produced by the HRs. The new optimized elicitation method was used to induce stilbenoid production in *A. ipaensis* and *A. duranensis* HRs, obtained in the same way as HRs of the peanut cv. Hull. The HRs of the wild relatives of peanut, upon elicitation synthesized less arachidin-1 and arachidin-3, but more arachidin-2 and arachidin-5 [194].

The HRs of *A. hypogaea* cv. Tainan9 were obtained by inoculation with *R. rhizogenes* strain K599 (NCPPB 2659). The production of stilbenoids in the roots was induced by the treatment of nine-day-old HRs with 100 µM MeJa in combination with 6.87 mM methyl-*β*-cyclodextrin. After 24 h of treatment, resveratrol (72.0 µg/g DW), arachidin-1 (179.3 µg resveratrol eq./g DW) and arachidin-3 (21 µg resveratrol eq./g DW) were found in the culture medium. MeJa applied alone did not induce the liberation of stilbenoids into the medium. Liquid chromatography with tandem mass spectrometry (LC-MS/MS) was applied to analyze the metabolites present in the spent medium from the elicited cultures. Except for the phenolic acids (hydroxybenzoic acid and caffeic acid), the following stilbenoids were detected in the medium; *trans*-piceatannol, *trans*- and *cis*-resveratrol, three isomers of *trans*-arachidin-1, 4-isopentadienyl-3,5,3’,4’-tetrahydroxystilbene, two isomers of *trans*-arachidin-3, two isomers of arahypin 7 and *trans*-3’-isopentadienyl-3,5,4’-trihydroxystilbene [196]. 

Another elicitation strategy was used with the HRs of *A. hypogaea* cv. Kalasin2 transformed with *R. rhizogenes* K599. Paraquat (PQ), a broad-spectrum herbicide that generates reactive oxygen species (ROS) in the plant tissue, was used in combination with MeJa and cyclodextrin. As was in the case of MeJa, PQ alone did not induce the liberation of stilbenoids from the roots. The most effective elicitation procedure that utilized 24 h of pretreatment with 500 µM PQ followed by the induction with 100 µM MeJa in combination with 6.87 mM methyl-*β*-cyclodextrin caused a sharp increase in stilbenoid biosynthesis. The elicited roots produced *trans*-resveratrol (1.3 mg/g DW, after 120 h), *trans*-arachidin-1 (180.1 mg/g DW, after 192 h) and *trans*-arachidin-3 (444.2 mg/g DW, after 120 h). LC-MS/MS analysis of the extract from the spent nutrient medium revealed the presence of hydroxybenzoic acid, two *trans*-piceatannol isomers, *trans*- and *cis*-resveratrol, jasmonic acid, four *trans*-arachidin-1 isomers, *trans*-arachidin-3, two isomers of arahypin 7 and arahypins 5 and 6 [197]. 

Wongshaya et al. [198] investigated an effect of mechanical stress (cutting) and light on stilbenoid biosynthesis in *A. hypogaea* cv. Tainan9 HRs. Mechanical stress increased the total phenolic content and antioxidant capacity of the cultures in both light and dark conditions. Roots cultivated in the dark showed a better response to elicitation than those grown in the light. Maximum yields of stilbenoids that were achieved using the elicitation procedure described earlier [197] were as follows: *trans*-resveratrol > 4 mg/g DW (uncut roots, dark, 72 h), *trans*-arachidin-1 ca. 240 mg/g DW (uncut roots, dark, 72 h), *trans*-arachidin-3 ca. 250 mg/g DW (uncut roots, dark, 72 h). The same elicitation procedure was applied to the *A. hypogaea* cv. Tainan9 HRs cultivated in a 5 L capacity stirred tank bioreactor or in 500 mL Erlenmayer flasks. Two densities of inoculum were evaluated: 5 g/L and 20 g/L. The optimum production of arachidins was achieved using 20 g/L inoculum grown in a 500 mL flask. Upon elicitation (72 h treatment), the HRs yielded ca. 1700 mg/L of *trans*-arachidin-1 and ca. 4800 mg/L of *trans*-arachidin-3. The roots grown in the bioreactor showed maximum productivity with 20 g/L inoculum, after 48 h treatment [199].

Three cultivars of peanut, including “Tifrunner,” “Hull,” and “Georgia Green” were transformed with *R. rhizogenes* ATCC 15843. The obtained HRs were treated with 125 µM MeJa, 18 g/L cyclodextrin, 3 mM H_2_O_2_ and 1 mM MgCl_2_ for 168 h. The extracts from the spent culture media were qualitatively and quantitatively analyzed to assess the content of stilbenoids. The HRs of the “Tifrunner” cultivar were found to be rich in arachidins 1, 2, 5 and 6, whereas the HRs of “Hull” cultivar preferably synthesized resveratrol and arachidin-3. The yields of the analyzed stilbenoids ranged from ca. 5 mg/L (arachidin-5 in “Georgia Green”) to ca. 170 mg/L (arachidin-1 in “Tifrunner”) [200].

Muscadine grape (*Vitis rotundifolia* Michx.), a grapevine species native to the southeastern part of North America, is a source of polyphenols that possess antioxidative and antimicrobial activity [201]. The HRs of *V. rotundifolia* (21 days old) were treated with 100 µM MeJa for 24 h. Extracts prepared from the root biomass and from the spent nutrient medium were subsequently analyzed in a search for stilbenoid metabolites. Resveratrol, piceid (resveratrol 3-*O*-glucoside) and ε-viniferin (resveratrol dehydrodimer) were present mainly in the biomass, whereas piceatannol was detected exclusively in the culture medium. Growth regulators IBA (0.05 mg/L) and BAP (0.05 mg/L) added to the culture medium did not affect yields of stilbenoids. Piceatannol and ε-viniferin were the best antioxidants among the analyzed HRs metabolites [202]. 

Ñopo-Olazabal et al. [203] used the HRs of muscadine grape to study the biochemical and molecular regulation of stilbenoid biosynthesis upon treatment with either 100 μM MeJA or with 10 mM H_2_O_2_, over a 96 h period. Both treatments induced the transcription of *PAL*, *STS* and resveratrol synthase gene (*RS*) as soon as 3 h after elicitation. Resveratrol, piceid, and ε-viniferin were identified in the control and in the elicited HRs. Except for resveratrol, the stilbenoids were accumulated in the roots. After the elicitation with MeJa, piceid content in the roots increased from 164 µg/g DW (0 h) to 337 µg/g DW at the end of experiment. The increase, however, was similar to that in the control roots. In the culture medium, piceid was undetectable until 12 h of the treatment and reached 4.7 µg/g DW at the end of the experiment. Maximum accumulation of resveratrol took place at 12 h after elicitation (106 µg/g DW) and then the content of the compound in the roots declined. The resveratrol content in the medium reached maximum at 18 h after exposition to MeJa (48 µg/g DW) and subsequently decreased to 6.4 µg/g DW at the 96 h. ε-Viniferin accumulated in the MeJa elicited roots up to 379 µg/g DW at the end of the experiment. The content was over twofold higher than that in the control roots. The culture medium contained up to 11.8 µg/g DW ε-viniferin. Hydrogen peroxide treatment was less effective as an inductor of stilbenoid biosynthesis. The piceid and resveratrol contents in the H_2_O_2_ treated roots increased to a lesser extent than it was observed for MeJa treatment. ε-Viniferin accumulated in the biomass (up to 434 µg/g DW, 96 h) and only ca. 5 µg/g DW of the compound was found in the nutrient medium. At 24 and 96 h after the treatment with H_2_O_2_, ca. 72.5 µg/g DW and 24.6 µg/g DW of resveratrol, respectively, were found in the nutrient medium. In the studied cultures, an increase in stilbenoid content correlated with an increased antioxidant capacity. The HRs of *V. vinifera* Pinot Noir cv. PN40024, obtained by inoculation with *R. rhizogenes* ATCC 15834 were cultivated in ½ SH (Schenk and Hildebrandt) medium supplemented with 2% sucrose. Piceid, resveratrol and two resveratrol dehydrodimers: ε-viniferin and δ-viniferin were constitutively present in the roots. δ-Viniferin was the major component of the stilbenoid fraction. The total stilbenoid content reached 217 µg/flask at the stationary phase of culture. The elicitation with MeJa (100 µM or 200 µM) on the 18th day of the culture (before the end of the exponential growth phase) increased an excretion rate of stilbenoids from 11% (control roots, after 10 days of the experiment) to 37% (200 µM MeJa, 10-day treatment). The control roots contained ca. 2.42 mg/g DW of stilbenoids at the end of experiment. The HRs elicited with 100 and 200 µM MeJa accumulated 6.98 mg/g DW and 4.34 mg/g DW of stilbenoids, respectively. The maximum stilbenoid content in the nutrient medium (19 mg/L) was found 10 days after the addition of MeJa (final concentration 200 µM). The concomitant use of MeJa (100 µM, added on the 18th day of culture) and methyl-*β*-cyclodextrins (30, 50 and 70 mM, added to the fresh culture medium before autoclaving) resulted in the increased stilbenoid contents in both root biomass and the culture medium. Maximum total stilbenoid contents, measured four days after the addition of jasmonate were 6.4 mg/g DW in the roots (30 mM cyclodextrins) and 165 mg/L in the medium (50 mM cyclodextrins). The combined treatment with MeJa and cyclodextrins caused enhanced liberation of stilbenoids (80–90%) into the nutrient medium [204]. Tisserant et al. [205], based on ^13^C and ^1^H NMR data and results of LC-MS analysis, identified major metabolites of *V. vinifera* Pinot Noir HRs. The main polyphenols found in the culture were stilbenoids (*trans*-resveratrol, *trans*-piceatannol, pallidol, ε-viniferin, scirpusin A, vitisin B and maackin) and flavanones (eriodictyol and naringenin).

Höll et al. [206] found that two R2R3-MYB-type transcription factors from grapevines regulated the stilbenoid biosynthetic pathway by the activation of the promoters of genes-encoding stilbene synthases (STS). One of the transcription factors, MYB15, expressed in the HRs of *V. vinifera* cv. Chardonnay increased levels of *STS* and *PAL* transcription. The content of *trans*-piceid in the *MYB15* transgenic HR lines was fivefold higher than that in the control roots. Accumulation of the remaining stilbenoids was less affected.

*V. vinifera* subsp. *sylvestris*, accessions W2 and W16 and cv. Rasha, were transformed using three different strains of *R. rhizogenes*: ArA4, Ar318 and LBA 9402. Although all the three bacterial strains induced HRs from the grapevine explants, HR lines obtained with the strain ArA4 showed the most vigorous growth. The highest resveratrol contents, 2 to 31 times higher than that found in the control roots, were found in the HRs obtained from internodal explants. The HR cultures originated from cv. Rasha, accession W2 and accession W16 contained up to 95, 256 and 273 µg/g DW of resveratrol, respectively. The elicitation with MeJa or sodium acetate caused approximately a twofold higher production of the stilbenoid and enhanced release of the product into the culture medium [207]

Ectopic expression of the genes-encoding enzymes and transcription factors engaged in stilbenoid biosynthesis and metabolism in the HRs of tobacco led to the production of biologically active stilbenoids. *V. vinifera* resveratrol-*O*-methyltransferase (VvROMT) and human cytochrome P450 hydroxylase 1B1 (HsCYP1B1) catalyze methylation of *trans*-resveratrol to *trans*-pterostilbene and hydroxylation of *trans*-resveratrol to *trans*-piceatannol. Tobacco leaf segments were inoculated with *R. rhizogenes* harboring the pRiA4 plasmid alone or pRiA4 with the binary plant expression vector pK7WG2_CYP1B1 or pJCN52_ROMT for the *HsCYP1B1* or *VvROMT* genes, respectively. The transgenic HRs expressing *CYP1B1* converted exogenously added *trans*-resveratrol into piceatannol, especially when treated with methyl-*β*-cyclodextrin as the permeabilizing agent. Biotransformation rates were: 0.4% in wildtype HRs and 1.4–1.6% in the transgenic HRs lines. In contrast to the transgenic HRs, piceid was the major biotransformation product in the wild-type HRs. The yields of piceatannol from the transgenic HR cultures carrying CYP1B1 and wild type HRs were 1888 µg/L and 819 µg/L, respectively. Addition of cyclodextrin to the medium may enhance the productivity up to 7 mg/L. Transgenic HRs carrying *VvROMT* metabolized exogenously added *trans*-resveratrol into *trans*-pterostilbene, piceid and piceatannol. Pterostilbene was found both in the roots and the culture medium. Maximum accumulation of the compound (2.6 µg/L) was observed 24 h after resveratrol feeding [208]. Tobacco HRs carrying *VvSTS* (gene for stilbene synthase from *V. vinifera*) and *AtMYB12* (gene for the transcription factor from *Arabidopsis thaliana*) produced over 450 µg/L stilbenoids and more flavonoids than the wild type HRs [8].

The *R. rhizogenes*-transformed roots of pigeon pea occurred to be an excellent source of cajaninstilbene acid, superior to seeds, leaves and roots of the parent plant [209]. The elicitation of HRs by 10 h of exposure to UV-B radiation resulted in over a twofold increased accumulation of cajaninstilbene acid (up to 6.6 mg/g DW). The radiation induced oxidative stress and caused damage to the roots, which, in turn, enhanced the production of signal molecule, salicylic acid, implicated in the regulation of the secondary metabolism biosynthetic pathways [134]. 

The HRs of pigeon pea induced by infection with *R. rhizogenes* strain K599 showed good biomass increase (17.3 g DW/L was obtained in 18 days of culture). The roots were elicited using the method described earlier for peanut HRs [194]. In 12-day-old cultures, the standard nutrient medium was replaced by the elicitation medium containing 125 µM MeJA, 18 g/L methyl-*β*-cyclodextrin, 3 mM H_2_O_2_ and 1 mM MgCl_2_ and the HRs were cultivated in the dark for another 7 days. After 144 h of treatment, the total content of cajaninstilbene acid in the culture reached over 8 mg/g DW. Almost 96% of the compound was secreted into the medium; thus, 7.7 mg/g DW of the product was extracted from the medium and the remaining 0.35 mg/g DW originated from the biomass. In the control roots, content of cajaninstilbene acid was 0.03 mg/g DW and the product could not be detected in the extract from the nutrient medium [210].

### 2.3. Hydrolyzable Tannins

**Figure 8:**
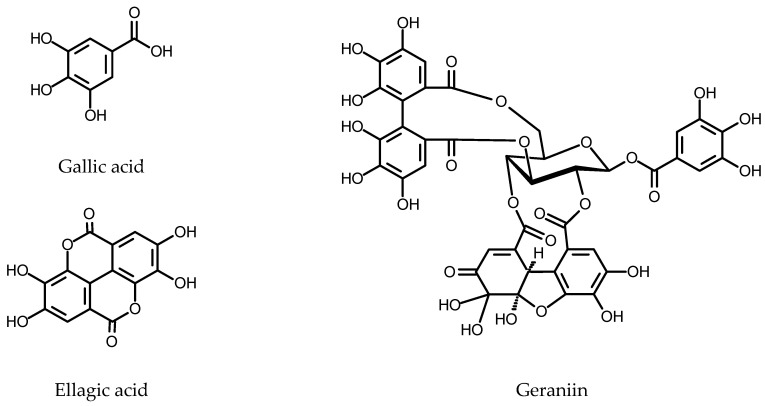

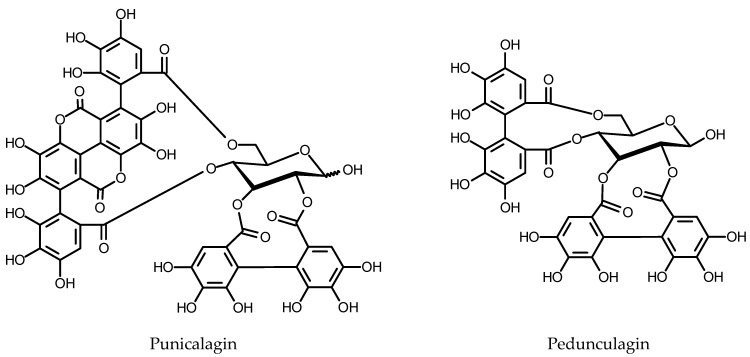
Chemical structures of gallic acid, ellagic acid and selected hydrolyzable tannins.

Plants that accumulate considerable amounts of hydrolysable tannins are traditionally used as anti-diarrhea medicines. They are also used topically as hemostatic, antiphlogistic and astringent agents. Hydrolyzable tannins (for structures, see Figure 8) are components of numerous food plants including the strawberry, raspberry, pomegranate and walnuts, but their bioavailability is poor. When ingested, hydrolyzable tannins are metabolized by gut microbiota to urolithins that are much better absorbed and are found in plasma mostly as glucuronides. The tannin metabolites have gained much interest as potential anticancer agents and estrogen receptor modulators of potential use in cardiovascular disorders, osteoporosis and several hormone-dependent diseases [211,212,213]. Hydrolyzable tannins production in the HRs has been studied chiefly to elucidate the regulation of the biosynthesis of these compounds.

*Sanguisorba officinalis* L. (Rosaceae) has been traditionally used as hemostatic and wound healing medicine. The HRs of the plant were obtained by inoculation with *R. rhizogenes* strain A4. Five phenolic compounds were identified in the investigated roots: gallic acid, 1,2,3,6-tetra-*O*-galloyl-*β*-D-glucose, 1,2,3,4,6-penta-*O*-galloyl-*β*-D-glucose, sanguiin H-6 and sanguiin H-11. Five lines of the studied HRs preferably synthesized sanguiin H-6 (0.217–0.569% FW) and one, showing the fastest growth, accumulated mainly 1,2,3,6-tetra-*O*-galloyl-β-D-glucose (0.322% FW) and sanguiin H-11 (0.221% FW). An intact plant contained 0.206% FW sanguiin H-6. The contents of hydrolysable tannins in the HRs were simililar to those found in adventitious roots; however, the biomass of HRs obtained in one four-week growth cycle was up to six times larger than that produced by adventitious roots [214].

The HRs of *Geranium thunbergii* Siebold ex Lindl. & Paxt. (Geraniaceae), transformed with *R. rhizogenes strain* A4 synthesized: gallic acid, ellagic acid, (+)-catechin, *β*-glucogallin (1-*O*-galloyl-*β*-D-glucopyranoside), 1,6-di-*O*-galloyl-*β*-D-glucose, 1,2,3,6-tetra-*O*-galloyl-*β*-D-glucose, 1,2,3,4,6-penta-*O*-galloyl-*β*-D-glucose, corilagin and geraniin. The HRs grown in ½ MS medium accumulated mainly 1,2,3,4,6-penta-*O*-galloyl-*β*-D-glucose whereas Gamborg’s B5 medium favored biosynthesis of geraniin [215]. *Phyllanthus niruri* L. (Phyllanthaceae) partly owes its medicinal properties to a high content of corilagin. The HRs of *P. niruri* produced neither corilagin nor geraniin but yielded several phenolic metabolites including gallic acid, (−)-epicatechin 3-*O*-gallate, (+)-gallocatechin and (−)-epigallocatechin 3-*O*-gallate [216]. *Fragaria ananassa* cv. Reikou infected by *R. rhizogenes* strain ATCC 15834 developed hairy roots that produced pedunculagin (2,3,4,6-tetra-*O*-galloyl-*β*-D-glucose). The tannin was present in the HRs grown in MS medium in the initial phase of growth (0.6% DW) and then its content declined (0.4% in 3-week culture). After 5 weeks of culture pedunculagin was not detected in the HRs. Two other nutrient media used in the study were less favorable for both biomass and pedunculagin production [35].

*Lawsonia inermis* L. (Lythraceae) is a source of henna, a pigment used to dye hair, skin and fingernails. A naphthoquinone, lawsone, is the compound responsible for dyeing properties as well as for bacteriostatic activity. Except for lawsone, gallic acid, flavonoids, coumarins and xanthones are known metabolites of the plant. The HRs of *L. inermis* were derived from the explants infected with *R. rhizogenes* strain NCIB 8196. The optimum growth of roots was achieved using MS medium. The HRs grown in the dark accumulated 1,2,3,6-tetra-*O*-galloyl-*β*-D-glucose (0.43% DW), 1,2,3,4,6-penta-*O*-galloyl-*β*-D-glucose (3.04% DW) and (+)-catechin. Normal roots contained up to 0.5% galloylglucoses, but up to 60 times more (+)-catechin [217].

*Punica granatum* L. (Lythraceae) cv. Wonderful was used to establish pomegranate HR culture. Three wild-type *R. rhizogenes* strains: MSU440, ATCC 15834 and A4, were transformed with a binary vector containing YFP (yellow fluorescent protein). The modified bacterial strains were used to initiate HRs. Punicalagin α and β were produced by the HRs with better efficiency than that achieved by in vitro cultured normal roots. The roots of the wild-type seedlings, however, contained more punicalagins. 

The glycosylation of gallic acid to *β*-glucogallin is the first step in punicalagin biosynthesis. The reaction is catalyzed by a specific UDP-glycosyltransferase (UGT). Transcriptomic analysis of pomegranate fruit peel (rich in tannins) allowed for the identification of 32 putative UGTs. By comparison with the results of transcript level analyses made for other tissues, two candidate genes were identified (*PgUGT1* and *PgUGT13*) that correlated with the tissue-specific abundance of tannins. To verify whether the two genes as well as the genes for the other UGTs are expressed in the HRs, RT-PCR was performed using RNA extracted from fruit peel, wild type roots and HRs. Expression of 26 putative UGTs was confirmed in all of the investigated tissue types [218]. Four candidate UGTs from pomegranate were cloned and biochemically characterized of which only two (UGT84A23 and UGT84A24) catalyzed formation of *β*-glucogallin. The overexpression or knockdown of the gene encoding one of the two UGTs in the HRs did not cause any obvious alterations in punicalagin accumulation. Double-knockdown HR lines accumulated less punicalagin but started to synthesize galloyl glucosides (ether-linked gallic acid and glucose). The new metabolites of the double-knockdown HR lines were gallic acid 3-*O*-glucopyranoside, gallic acid 4-*O*-glucopyranoside and digalloyl glucose conjugate. The results suggested that in the HRs, gallic acid may be utilized by an unknown UGTs for glucoside formation [219]. In a search for the UGTs engaged in the synthesis of gallic acid glycosides 11 candidate UGTs were identified by the comparison of UGTs expression in control root lines and double knockdown lines of pomegranate HRs. Of the 11 candidate UGTs, only one was active toward gallic acid and catalyzed the formation of a single product: gallic acid 4-*O*-glucoside [220].

## 3. Conclusions

Table 1 summarizes the most spectacular achievements of the research on highly productive hairy root cultures or genetically modified plants containing polyphenolic antioxidants from the flavonoid, stilbenoid and hydrolyzable tannin group. The high yields of the polyphenolic products were obtained using procedures like optimization of culture conditions, selection of the plant material and bacterial strain used to initiate the HRs, elicitation, permeabilization of plant tissue, adding the enzyme cofactors and genetic engineering (overexpression, heterologous expression or silencing of genes-encoding the regulatory transcription factors and biosynthetic enzymes). It should be considered, however, that high contents of polyphenols in the HRs may be not accompanied by the vigorous root growth. This can hamper applications of the cultures that accumulate products in the cells. 

HR cultures provide a valuable tool for the rapid characterization of regulatory mechanisms in plant polyphenol biosynthesis, and elucidation of the roles played by the particular biosynthetic enzymes or transcription factors. The use of HRs has led to the identification and description of several enzymes implicated in polyphenol biosynthesis. Nowadays, it seems that HR culture is primarily a useful tool in the genetic engineering of plants. The implementation of the industrial process using HRs to produce antioxidant natural products looks like a distant prospect.

## Figures and Tables

**Table 1:** Hairy root cultures that produced high yields of antioxidative natural products.

Plant Species	Hairy Roots	Natural Product	Maximum Content in the Biomass	Lit.
*Fragaria x ananassa Duch* cv. *Reikou*	Wild type	Procyanidin B-3	8 mg/g DW	[35]
*Litchi chinensis* Sonn.	Overexpressing *LcMYB1*	Anthocyanins Proanthocyanidins	3 mg/g FW 15 mg/g FW	[48]
*Vitis vinifera* L.	Overexpressing *VvMybP1*, *VvMybP2*	Proanthocyanidins	8 mg/g FW	[38]
*Fagopyrum tataricum* (L.) Moench. cv. Hokkai T10	Wild type	Cyanidin 3-*O*-glucoside Cyanidin 3-*O*-rutinoside	0.8 mg/g DW 2.4 mg/g DW	[52]
*Daucus carota* L. ssp. *sativus* var. atrorubens Alef	Wild type, treated with ethephon	Anthocyanins	8 mg/g DW	[9]
*Antirrhinum majus* L.	Overexpressing *AmRosea1*	Anthocyanins	2 mg/g FW	[58]
*Fagopyrum esculentum* Moench	Wild type	(−)-Epicatechin 3-O-gallate (+)-Catechin	10 mg/g DW 8 mg/g DW	[61]
*Fagopyrum tataricum* (L.) Moench cv. Hokkai T10	Wild type	Rutin	59 mg/g DW	[34]
*Fagopyrum tataricum*	Overexpressing *FtUGT73BE5*	Rutin	Up to 80 mg/g DW	[65]
*Polygonum multiflorum* Thunb.	Wild type, elicited with MJ (50 µM)	Quercetin	Up to 14 mg/g DW	[74]
*Isatis tinctoria* L.	Wild type, exposed to UV-B	Rutin Quercetin Isorhamnetin Kaempferol	Up to 1.5 mg/g DW Up to 1.7 mg/g DW Up to 2.0 mg/g DW Up to 2.2 mg/g DW	[84]
*Ligularia fischeri* (Ledeb.) Turcz.	Wild type	Myricetin	2.4 mg/g DW	[86]
*Scutellaria baicalensis* Georgi	Wild type	Baicalein derivatives Wogonin derivatives Oroxylin A derivatives	68.7 mg/g DW 15.1 mg/g DW 11.9 mg/g DW	[96]
*Scutellaria baicalensis* Georgi	Overexpressing *SbPAL*	Baicalin Baicalein Wogonin	136 mg/g DW 29 mg/g DW 6.7 mg/g DW	[102]
*Scutellaria lateriflora* L.	Wild type	Baicalin Wogonoside Wogonin	14.5 mg/g DW 12.0 mg/g DW 11.5 mg/g DW	[111]
*Saussurea medusa* Maxim	Wild type	Jaceosidin	6.1 mg/g DW	[126]
*Saussurea involucrata* Kar. et Kir. ex Maxim	Overexpressing *SmCHI*	Apigenin	2.6 mg/g DW	[127]
*Erigeron breviscapus* (Vaniot) Hand.-Mazz.	Overexpressing *EbCHI*	Scutellarin	2.2 mg/g DW	[128]
*Glycine max* (L.) Merr.	Overexpressing *GmMYB205*	Daidzein derivative	6.7 mg/g DW	[141]
*Glycine max* (L.) Merr.	Overexpressing *GmMaT2*	Malonyldaidzin	6.3 mg/g FW	[145]
*Glycine max* (L.) Merr.	Wild type, elicited with MeJa (100 µM)	Daidzin	33.9 mg/g DW	[146]
*Trifolium pratense* L.	Wild type	Daidzein Genistein Formononetin Biochanin A	8.6 mg/g DW 2.5 mg/g DW 15.2 mg/g DW 1.1 mg/g DW	[149]
*Astragalus membranaceus* (Fisch.) Bunge	Wild type, co-cultivated with the immobilized *Aspergillus niger*	Calycosin Formononetin	0.7 mg/g DW 1.1 mg/g DW	[155]
*Psoralea lachnostachys* F. Muell	Wild type	Daidzein Coumestrol	10.2 mg/g DW 0.5 mg/g DW	[158]
*Psoralea corylifolia* L.	Wild type	Daidzin	Over 10 mg/g DW	[159]
*Pueraria candollei* Wall. Ex Benth	Wild type	Daidzin Puerarin	Up to 29.9 mg/g DW Up to 3.4 mg/g DW	[164]
*Ononis spinosa* L.	Wild type	Medicarpin glucoside Sativanone glucoside Pseudobaptigenin glucoside	22.3–28.9 mg/g DW 5.6–11.4 mg/g DW 1.2–2.0 mg/g DW	[170]
*Arachis hypogaea* L. cv. Hull	Wild type, elicited with MeJa (100 µM) and cyclodextrin (9 g/L)	Resveratrol Piceatannol Arachidin-1 Arachidin-3	5.3 mg/g DW 0.3 mg/g DW 4.0 mg/g DW (56 mg/L) 17.1 mg/g DW (148 mg/L)	[190]
*Arachis hypogaea* L. cv. Hull	Wild type, elicited with MeJa (125 µM), cyclodextrin (18 g/L), H_2_O_2_ (3 mM), and MgCl_2_ (1 mM)	Arachidin-1 Arachidin-2 Arachidin-3 Arachidin-5	227.4 mg/L 83.1 mg/L 370.6 mg/L 68.4 mg/L	[194]
*Arachis hypogaea* L. cv. Kalasin2	Wild type, elicited with paraquat (500 µM), MeJa (100 µM) and cyclodextrin (6.87 mM)	Resveratrol Arachidin-1 Arachidin-3	1.3 mg/g DW 180.1 mg/g DW 444.2 mg/g DW	[197]
*Arachis hypogaea* L. cv. Tainan9	Wild type, elicited with paraquat (500 µM), MeJa (100 µM) and cyclodextrin (6.87 mM)	Arachidin-1 Arachidin-3	1700 mg/L 4800 mg/L	[199]
*Vitis rotundifolia* Michx.	Wild type, elicited with MeJa (100 µM)	Piceid ε-Viniferin	0.34 mg/g DW 0.38 mg/g DW	[203]
*Vitis rotundifolia* Michx	Wild type, elicited with H_2_O_2_ (10 mM)	ε-Viniferin	0.43 mg/g DW	[203]
*Vitis vinifera* Pinot Noir cv. PN40024	Wild type, elicited with MeJa (100 µM)	Total stilbenoids	6.98 mg/g DW	[204]
*Vitis vinifera* Pinot Noir cv. PN40024	Wild type, elicited with MeJa (100 µM) and cyclodextrin (50 mM)	Total stilbenoids	165 mg/L (medium) 6.4 mg/g DW	[204]
*Vitis vinifera* subsp. *sylvestris* acc. W16	Wild type	Resveratrol	0.27 mg/g DW	[207]
*Cajanus cajan* (L.) Millsp.	Wild type, irradiated with UV-B	Cajaninstilbene acid	Up to 6.6 mg/g DW	[134]
*Cajanus cajan* (L.) Millsp.	Wild type, elicited with MeJa (125 µM), cyclodextrin (18 g/L), H_2_O_2_ (3 mM), and MgCl_2_ (1 mM)	Cajaninstilbene acid	Over 8 mg/g DW	[210]

## Data Availability

Not applicable.

## Abbreviations

4CL	4-coumarate:CoA ligase (4-coumaroyl CoA ligase)
ANR	anthocyanidin reductase
ANS	anthocyanidin synthase
BAP	6-benzylaminopurine
bHLH	basic helix-loop-helix
BHT	butylohydroxytoluen
C4H	cinnamate 4-hydroxylase
CHI	chalcone isomerase
CHS	chalcone synthase
DFR	dihydroflavonol 4-reductase
DW	dry weight
F3H	flavone 3-hydroxylase
F3*β*OH	flavanone 3-*β*-hydroxylase
F6H	flavone 6-hydroxylase
F8H	flavone 8-hydroxylase
FNS	flavone synthase
FW	fresh weight
GUS	*β*-glucuronidase
HPLC-PAD-ESI-MS^n^	high-performance liquid chromatography with photodiode detection, electrospray ionization, and multi-stage mass spectrometry
HR	hairy roots
IBA	indole-3-butyric acid
IF7Mat	isoflavone 7-*O*-glucoside-6”-*O*-malonyltransferase
IFD	isoflavone dehydratase
IFS	isoflavone synthase
IOMT	isoflavone-*O*-methyltransferase
LAR	leucoanthocyanidin reductase
LC-MS/MS	liquid chromatography with tandem mass spectrometry
MaT	malonyl CoA:flavonoid acyltransferase
MeJa	methyl jasmonate
MS	Murashige and Skoog
PA	proanthocyanidin
PAL	phenylalanine ammonia lyase
*PAP1*	production of anthocyanin pigment 1 gene
PQ	paraquat
RS	resveratrol synthase
SA	salicylic acid
SH	Schenk and Hildebrandt
STS	stilbene synthase
TOF-MS	time-of-flight mass spectroscopy
TT2	transparent testa 2 transcription factor
UBGT	UDP-glucose:baicalein 7-*O*-glucosyltransferase
UGT	UDP-glucosyltransferase
UHPLC-PDA	ultra-high-performance liquid chromatography with photodiode detection
YE	yeast extract
